# Network of protein interactions within the *Drosophila* inner kinetochore

**DOI:** 10.1098/rsob.150238

**Published:** 2016-02-24

**Authors:** Magdalena M. Richter, Jaroslaw Poznanski, Anna Zdziarska, Mariusz Czarnocki-Cieciura, Zoltan Lipinszki, Michal Dadlez, David M. Glover, Marcin R. Przewloka

**Affiliations:** 1 Department of Genetics, University of Cambridge, Cambridge, UK; 2 Institute of Biochemistry and Biophysics, Polish Academy of Science, Warsaw, Poland; 3 Institute of Genetics and Biotechnology, Faculty of Biology, University of Warsaw, Warsaw, Poland

**Keywords:** chromosome, HDX-MS, kinetochore, centromere, mitosis, structure

## Abstract

The kinetochore provides a physical connection between microtubules and the centromeric regions of chromosomes that is critical for their equitable segregation. The trimeric Mis12 sub-complex of the *Drosophila* kinetochore binds to the mitotic centromere using CENP-C as a platform. However, knowledge of the precise connections between Mis12 complex components and CENP-C has remained elusive despite the fundamental importance of this part of the cell division machinery. Here, we employ hydrogen–deuterium exchange coupled with mass spectrometry to reveal that Mis12 and Nnf1 form a dimer maintained by interacting coiled-coil (CC) domains within the carboxy-terminal parts of both proteins. Adjacent to these interacting CCs is a carboxy-terminal domain that also interacts with Nsl1. The amino-terminal parts of Mis12 and Nnf1 form a CENP-C-binding surface, which docks the complex and thus the entire kinetochore to mitotic centromeres. Mutational analysis confirms these precise interactions are critical for both structure and function of the complex. Thus, we conclude the organization of the Mis12–Nnf1 dimer confers upon the Mis12 complex a bipolar, elongated structure that is critical for kinetochore function.

## 1. Introduction

Kinetochores are multiprotein supramolecular assemblies responsible for connecting microtubules (MTs) to chromosomes and hence are critical for proper chromosome segregation [1]. The kinetochore not only provides this physical linkage but is also endowed with spring-like properties that enable it to respond to tension that through the spindle assembly checkpoint (SAC) can signal and correct improper MT attachments. It also acts as a platform for the loading of the SAC proteins and of motor and other MT-associated proteins that regulate behaviour of the MT plus ends and chromosome movement (reviewed in [2–5]). To fully understand these multiple properties, it is necessary to obtain precise structural information of how the kinetochore is built.

The kinetochore comprises a super-complex known as the KMN network (composed of the KNL1, Mis12 and Ndc80 complexes) that directly links centromeres to MTs and constitutes a binding platform for many other kinetochore proteins, including SAC components [6,7]. The Mis12 complex (Mis12C) is first to assemble on centromeres by direct binding to the centromeric protein CENP-C to constitute the foundation upon which the mitotic kinetochore is constructed [8,9]. In vertebrate cells, CENP-C is one member of the constitutive centromere-associated network (CCAN) that maintains centromeric identity and enables kinetochore formation. The CCAN, currently thought to comprise 16 proteins in human cells, associates with centromeres throughout most of the cell cycle. It is composed of a number of sub-complexes recognized for their distinct functions [3,10,11]. In contrast, multiple proteomic studies have led to the conclusion that organisms such as *Drosophila melanogaster* or *Caenorhabditis elegans* have a single CCAN component, namely CENP-C [12,13]. Thus CENP-C is critical for kinetochore assembly in these species. The amino-terminal part of CENP-C binds directly to kinetochore components and provides the key link between the centromere and the mitotic kinetochore [14,15]. In *Drosophila*, the centromeric histone CENP-A/CID together with its loading factor CAL1, and CENP-C are mutually dependent for their centromeric association and kinetochore formation [16–18]. This essential role of CENP-C as a platform for the Mis12C in kinetochore assembly is conserved among different organisms, including humans [15,19].

Proteomic studies have revealed that the *Drosophila* Mis12C consists of three subunits, Mis12, Nnf1 and Nsl1 [20,21]; in contrast its vertebrate counterpart has a fourth component, Dsn1 [22–24]. The three subunits of the *Drosophila* Mis12C are co-dependent for their localization to mitotic kinetochores [20]. They are recruited to kinetochores during prophase at roughly the same time and this process is contemporaneous with the centromeric loading of the large KMN network component Spc105/KNL1 [25]. Depletion of any of the Mis12C subunits leads to similar chromosome missegregation defects [26]. Because binding of the Mis12C to the centromeric CENP-C is the very first step of the kinetochore assembly and the components of this complex are located in close proximity to the mitotic centromeres, Mis12C may be considered as the foundation of the mitotic kinetochore [22,27].

It has been difficult to relate the function of the Mis12C to structure because currently crystallographic studies of the Mis12C of any organism have not proved possible and existing studies have been limited to the use of chemical cross-linking of its subunits and electron microscopy [23,28,29]. In order to gain insight into the organization of the *Drosophila* Mis12C and to define the surfaces of interactions of its proteins with each other and with CENP-C, we have employed hydrogen–deuterium exchange combined with mass spectrometry (HDX-MS). We were then able to use mutational analysis to test the requirements of individual residues within the identified interacting motifs for proper binding between subunits and with CENP-C. It emerged that in *Drosophila* Mis12 and Nnf1 form an elongated heterodimer that provides a base for all other interactions within the Mis12 complex. The dimer is stabilized by the coiled-coil (CC) interactions within the carboxy-termini of Mis12 and Nnf1. Moreover, adjacent to this dimerization motif we found the interaction site for another Mis12C component, Nsl1. The amino-termini of Mis12 and Nnf1 bind to the helical domain of CENP-C located near its N-terminus. Together, our findings describe the organization and protein–protein interaction network within the Mis12C which defines the basis of this crucial linkage between the kinetochore and centromere.

## 2. Results

### 2.1. Nnf1a and Mis12 interact via their carboxy-terminal coiled-coil domains

**Figure 1.**
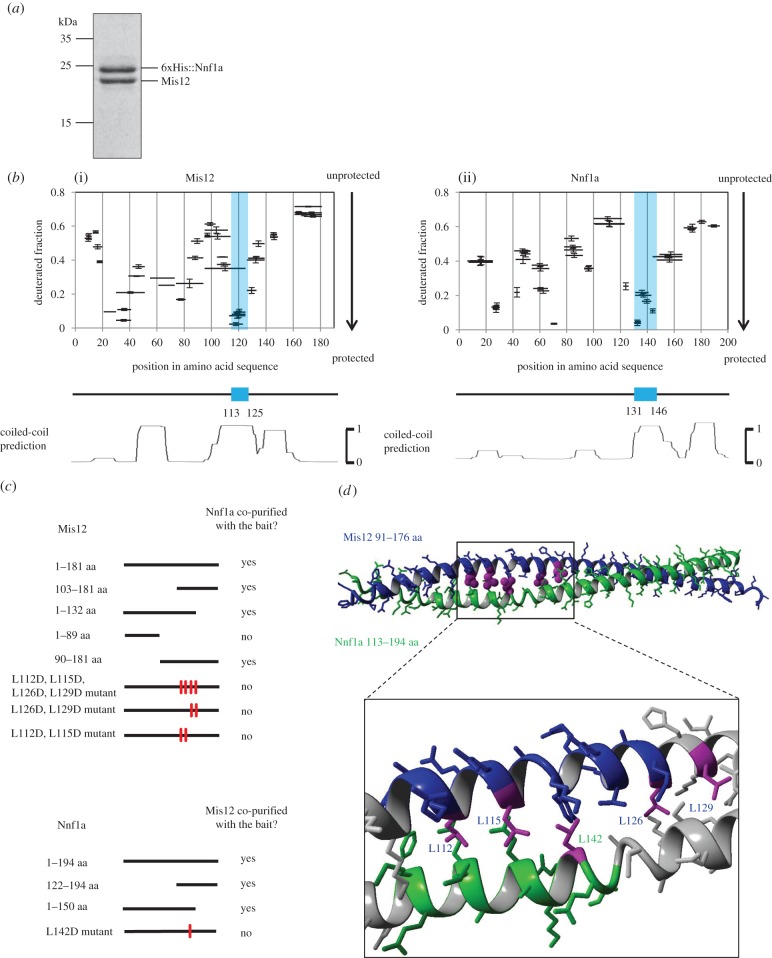
Nnf1a and Mis12 interact via C terminal coiled-coil (CC). (*a*) CBB-stained SDS-PAGE gel showing Mis12–Nnf1a complex after co-expression, co-purification and size-exclusion chromatography (SEC). Mis12 co-purifies with 6xHis::Nnf1a when co-expressed in *E. coli* using the Duet vector system. Identity of both proteins was confirmed by western blotting (WB) and MS analysis. (*b*) Patterns of hydrogen–deuterium exchange in Mis12 (i) and Nnf1a (ii) when in complex revealing protected (i.e. structured) regions. Graphs represent fractional deuteration levels of Mis12 and Nnf1a peptides after 10 s of HDX. Black bars represent peptides identified by MS. The *X*-axis indicates their position in amino acid sequence. The *Y*-axis shows relative deuterium uptake calculated as described in Material and methods. Value 1 is considered to be maximal deuteration level, meaning that all hydrogens in amide bonds of particular peptide were exchanged to deuterium. Regions that were most protected in the carboxy-termini of Mis12 and Nnf1a are indicated by blue rectangles. Error bars represent standard deviation, calculated based on triplicate experiments. Lower panels show CC predictions, aligned to match amino acid sequence on the *X*-axis, with the most protected region coincident with one of the predicted coils. CC predictions were calculated with the program COILS [32]. (*c*) Schematic of the affinity purification–MS (AP-MS) results from D.mel-2 cultured cells. Mis12 and Nnf1a truncations and mutants were fused with GFP and used as baits. MS analysis of AP eluates was used to confirm the recruitment of the partner of the complex by given bait. Thick black lines correspond to the length of particular bait construct. Red marks indicate mutated amino acids. Detailed information about the number of proteins' peptides identified by MS and their scores can be found in the electronic supplementary material, table S1. (*d*) Model of Mis12–Nnf1a heterodimer built using the crystal structure of the CC domain of *C. elegans* SAS-6 (pdb record 4GKW) as a template. Leucines that were mutated in AP-MS and yeast-2-hybrid (Y2H) experiments are marked in magenta. Black box indicates fragments that are involved in direct interaction, based on NMR experiments.

The precise nature of the intermolecular interactions between the subunits of the Mis12 complex (Mis12C) is not known beyond the findings that its Nnf1a and Mis12 components directly interact with each other in the yeast 2-hybrid (Y2H) assay [30,31] and that they form a dimer *in vitro* [23,28,29]. However, the exact regions of the physical interactions, either *in vivo* or *in vitro*, have not been defined. In order to narrow down the interaction domains and then gain insight into the structure of both proteins, we first co-expressed recombinant *Drosophila* Nnf1a and Mis12 in *Escherichia coli*, and affinity purified the dimer on Ni-NTA agarose beads followed by size-exclusion chromatography (figure 1*a*). To this end, we used the bacterial Duet system, which allows the co-expression of multiple different proteins, of which one may be tagged. This permits one-step affinity purification of bait together with its interacting partners. Indeed, we found that Mis12 and Nnf1a formed a heterodimer (MN dimer), which we could co-purify to homogeneity using polyhistidine (His)-tagged Nnf1a as bait. The Mis12–Nnf1a complex was very stable in solution, in contrast to the single subunits, which underwent rapid degradation when expressed individually. For this reason, we were unable to study Mis12 and Nnf1a proteins separately (data not shown). The purified heterodimer was subsequently analysed by HDX-MS to identify regions protected from hydrogen–deuterium (H/D) exchange. Since the exchange may be retarded for amide protons localized in interacting regions, such data are indicative of the localization of the interacting surfaces within the dimeric complex. We observed varying levels of protection from exchange in both proteins, with some regions exhibiting much slower exchange than others (figure 1*b*). The most prominent regions of protection were approximately 15 amino acids in length (blue regions in figure 1*b*) located around residue 140 in Nnf1a and around residue 120 in Mis12. Alignment of the predicted CC regions with the fractional deuteration distribution revealed that the highly protected regions precisely matched one of the predicted CC structures (figure 1*b*, lower panels). This suggested that Nnf1a and Mis12 might bind each other via interactions of these CC domains in their carboxy-terminal regions. To test this hypothesis, we generated and expressed a set of truncated Nnf1a and Mis12 DNA constructs fused to GFP in cultured *D. melanogaster* D.mel-2 cells. We then performed GFP-trap affinity purifications followed by mass spectrometric (MS) analysis (AP-MS) (figure 1*c* and electronic supplementary material, table S1) to reveal their interacting partners. Truncated forms of Nnf1a containing residues 122–150 were able to pull down Mis12 protein and truncations of Mis12 retaining amino acids 103–132 could pull down Nnf1a. These fragments correspond well to those regions of Nnf1 and Mis12 found to be interacting by HDX-MS (residues 131–146 and 113–125, respectively), thus providing further confirmation of the physiological importance of the identified interacting surfaces. Another amino-terminal fragment of Mis12 (residues 1–89), which contains a different region indicated by HDX-MS as potentially interacting with Nnf1a, did not co-purify endogenous Nnf1 protein from cultured *Drosophila* cells, leading us to conclude that the protection of this region within the Mis12–Nnf1a dimer is not a consequence of direct interactions between the two proteins. Similarly, the region 25–45 aa in Nnf1a, which also showed an increased level of protection in HDX-MS experiments, can be excluded as necessary for interaction with Mis12. This conclusion is based on AP-MS experiments using Nnf1a^L142D^ mutant as bait (see below). This mutant although containing the 25–45 aa region failed to pull down Mis12 protein from D.mel-2 cell extracts. In conclusion, the CC regions of both Mis12 and Nnf1a proteins were found to be involved in the direct binding between these two proteins in HDX-MS and in *in vivo* and *in vitro* pull-down experiments. Amino-terminal regions, although protected in the HDX-MS, proved to be insufficient for supporting this interaction as shown by the binding assays.

These findings led us to perform computational modelling of the tertiary structure of carboxy-termini of Nnf1a and Mis12 within the heterodimer. We used the crystal structure of the CC domain of *C. elegans* SAS-6 (PDB record 4GKW) as a template for such modelling. For each protein, we tested 60 various alignments, differing iteratively by a single residue shift. This resulted in the model presented in figure 1*d*, which was selected from 3600 trials as the one displaying the largest number of leucine–leucine contacts (for details see Material and methods and electronic supplementary material, figure S1). Based on this model, we synthesized two peptides (Nnf1a^122–147^ and Mis12^101–130^) and performed circular dichroism (CD) measurements (electronic supplementary material, figure S2). These confirmed the existence of the *α*-helical structure of the peptides and the presence of intermolecular interactions stabilizing the Mis12–Nnf1 peptide complex. We further analysed the dimer of the peptides using nuclear magnetic resonance (NMR). A 2D-TOCSY spectrum recorded for the mixture of peptides showed shift in the location of resonances assigned to residues ^130^F-I^144^ of Nnf1a^122–147^ and ^109^T-L^126^ of Mis12^101–130^, including six out of seven leucine residues, potentially involved in the formation a CC structure, thus identifying the putative dimerization interface (electronic supplementary material, figure S3).

In order to disrupt the CC and the interaction surface between Nnf1a and Mis12, we replaced the critically important leucine residues with aspartic acid by *in vitro* mutagenesis. We then established stable D.mel-2 cell lines expressing GFP-tagged full-length Nnf1a or Mis12 with the appropriate mutations. A single point mutant Nnf1a^L142D^ was unable to pull down Mis12 in the AP-MS study (figure 1*c*). It also failed to interact with Mis12 in a Y2H assay (electronic supplementary material, table S2). Similar results were obtained for Mis12 mutants (Mis12^L112D,L115D,L126D,L129D^, Mis12^L112D,L115D^ in AP-MS; Mis12^L126D,L129D^ in AP-MS and Y2H; Mis12^L112D^, Mis12^L115D^, Mis12^L126D^, Mis12^L129D^ in Y2H). Together, these data indicate that a direct interaction between Nnf1a and Mis12 occurs via the regions identified by the HDX-MS as highly protected from H/D exchange (Nnf1a^131–146^ and Mis12^113–125^) and that these regions indeed form a CC structure.

### 2.2. Mis12–Nnf1a forms the interaction platform for CENP-C and Nsl1

**Figure 2.**
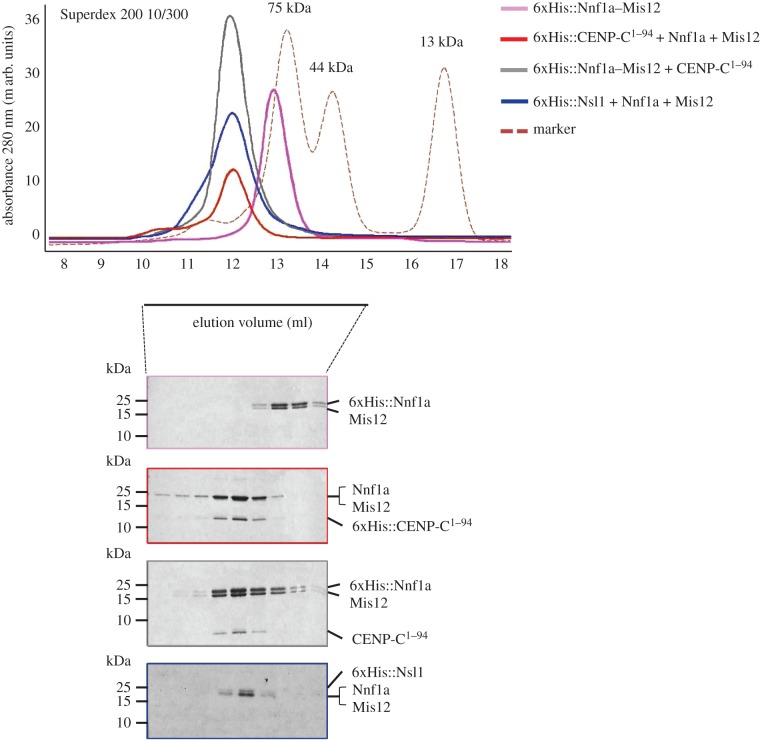
Mis12–Nnf1a–CENP-C^1–94^ and Mis12–Nnf1a–Nsl1 form elongated complexes. SEC of recombinant protein complexes co-expressed in *E. coli* using the Duet vector system with corresponding CBB-stained SDS-PAGE of SEC fractions. Complex formation is indicated by the shift in the elution profile of CENP-C^1–94^ and Mis12 + Nnf1a as well as Nsl1 and Mis12 + Nnf1a in comparison with Nnf1a + Mis12. Red dashed line represents elution profile of SEC protein markers: conalbumin (75 kDa), ovalbumin (44 kDa) and ribonuclease (13 kDa). 6xHis::Nnf1a + Mis12 + CENP-C^1–94^ complex and 6xHis::CENP-C^1–94^ + Nnf1a + Mis12 elute at the same volume, which proves that the 6xHis tag does not influence the complex formation nor the complex general structure. On SDS-PAGE, untagged Mis12 and Nnf1a tend to migrate in the same band. Presence of both proteins was confirmed by MS analysis.

According to published studies, the amino-terminal part of human CENP-C binds directly to the Mis12C [15]. Our AP-MS data have also revealed that the first 94 amino acids of *Drosophila* CENP-C are sufficient to pull down all the KMN network components, including Mis12C, from an extract of D.mel-2 cells (electronic supplementary material, table S1). We also know that another Mis12C subunit, protein Nsl1, binds to Mis12 and Nnf1, but the details of this interaction have remained obscure. In order to map the interacting surfaces between Mis12, Nnf1a, Nsl1 and CENP-C, we carried out two types of experiment. In the first, we expressed residues 1–188 of CENP-C and attempted to demonstrate binding to members of the Mis12C in Y2H assays. In the second, we co-expressed residues 1–94 of CENP-C with Mis12C components in the Duet system followed by co-purifications. We did not observe any direct pairwise interactions between CENP-C fragments and individual Mis12C proteins. Nor, in similar experiments, were we able to identify any interactions between either Mis12 or Nnf1a and Nsl1 (electronic supplementary material, figure S4). Although we managed to express and purify Mis12, Nnf1a and Nsl1 on their own in small amounts sufficient to be visualized by immunoblotting (electronic supplementary material, figure S4b), we failed to obtain higher yields due to either insolubility or rapid degradation (data not shown). It is therefore possible that the lack of observable interactions between individual subunits *in vitro* is due to an insufficient amount of stable protein. In order to provide an alternative explanation which could be examined experimentally, we hypothesized that interaction surfaces formed by more than two proteins could be required for complex formation. To test this hypothesis, we performed co-expression of multiple Mis12C subunits with CENP-C fragments in different combinations using His-tags fused to different subunits to achieve co-purification. In the course of these experiments, we found that the amino-terminal part of CENP-C (residues 1–94) formed a stable complex and co-purified with Mis12 and Nnf1a; Nsl1 was also able to bind efficiently to the Mis12–Nnf1a dimer (figure 2).

To characterize the size, shape and stoichiometry of purified complexes, we performed size-exclusion chromatography (SEC) combined with multi-angle light scattering (MALS). SEC separates proteins based on size and shape, whereas MALS provides the information on the molecular mass of the intact complexes. We first compared SEC profiles of Mis12–Nnf1a (MN), Mis12–Nnf1a–CENP-C^1–94^ (MN-CC) and Mis12–Nnf1a–Nsl1 (MN-N) complexes (figure 2). MN-CC and MN-N migrated faster through a Superdex 200 SEC column than MN, confirming that both CENP-C^1–94^ and Nsl1 can form stable complexes with the MN dimer. All three complexes eluted from the column earlier than expected based on their predicted molecular weights, which suggests that they may adopt an elongated ellipsoid structure. These results are in accord with earlier studies of the human [23,33] and yeast [28,29,34] Mis12 complexes. Together with these previous reports, our data indicate that Mis12 complexes adopt an elongated shape due to the rod-like organization of the MN dimer.

Molecular masses calculated by MALS correspond to theoretical masses of complexes in which each subunit is represented once, thus indicating that the subunits were present at stoichiometry of 1 : 1 (in the case of MN) and 1 : 1 : 1 (in the case of MN-CC and MN-N) (table 1 and electronic supplementary material, figure S5).

**Table 1.** Stoichiometry of Mis12C components: comparison of theoretical and MALS-calculated masses of studied complexes.

complex	theoretical mass of the complex (kDa)	mass calculated by MALS (kDa)	stoichiometry
6xHis::Nnf1a + Mis12	43.378	46.62	1 : 1
6xHis::Nnf1a + Mis12 + CENP-C^1–94^	56.367	56.73	1 : 1 : 1
6xHis::CENP-C^1–94^ + Nnf1a + Mis12	56.525	56.12	1 : 1 : 1
6xHis::Nsl1 + Nnf1a + Mis12	67.424	72.02	1 : 1 : 1

Additionally, we observed that MN and MN-CC complexes tend to form oligomers. Protein complexes that were already fractionated and collected from the SEC column and stored at 4°C in SEC buffer formed oligomeric structures. These were re-loaded and fractionated on an SEC column again revealing two distinct elution peaks (electronic supplementary material, figure S6). MALS molecular mass calculations revealed that the complex present in the first, faster migrating peak was twice the mass of the one in the second peak, which suggests that CENPC^1–94^–Mis12–Nnf1a and Mis12–Nnf1a assemblies tend to form oligomeric (perhaps heterodimeric) structures.

### 2.3. Nsl1 binds carboxy-terminal fragments of Nnf1a and Mis12

**Figure 3.**
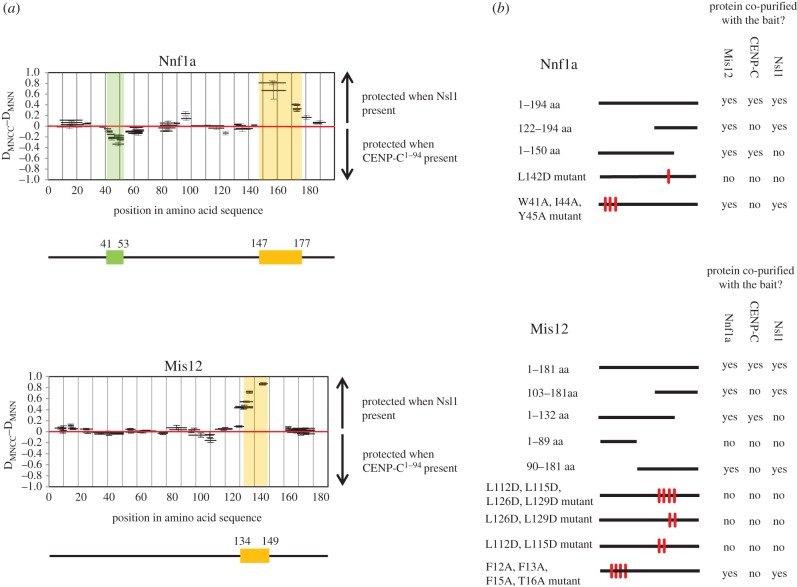
Interaction region between Mis12–Nnf1a complex and Nsl1 or CENP-C. (*a*) Difference in fractional deuterium uptake after 10 s of HDX for Nnf1a (upper panel) and Mis12 (lower panel) peptides between two ternary complexes, one, Mis12–Nnf1a–CENP-C^1–94^ and second, Mis12–Nnf1a–Nsl1. Thick black bars represent peptides identified by MS. All the peptides above the red line are better protected from the HDX when Mis12–Nnf1a is in complex with Nsl1. All the peptides below the red line are better protected from the HDX when Mis12–Nnf1a is in complex with CENP-C^1–94^. Regions protected the most when in complex with Nsl1 are indicated by orange boxes. Regions protected the most when in complex with CENP-C^1–94^ is indicated by the green box. Error bars represent standard deviation, calculated as the square root of the sum of variances of the subtracted numbers, based on triplicate experiments. (*b*) Schematic of AP-MS results from D.mel-2 cultured cells. Nnf1a and Mis12 truncations and mutants were fused with GFP and used as baits. MS analysis of AP eluates was used to confirm the recruitment of the partner of the complex by the given bait. Black lines correspond to the length of the particular bait construct. Red marks indicate mutated amino acids. Detailed information about numbers of the baits' and preys' peptides identified by MS and their scores can be found in the electronic supplementary material, table S1.

**Figure 4.**
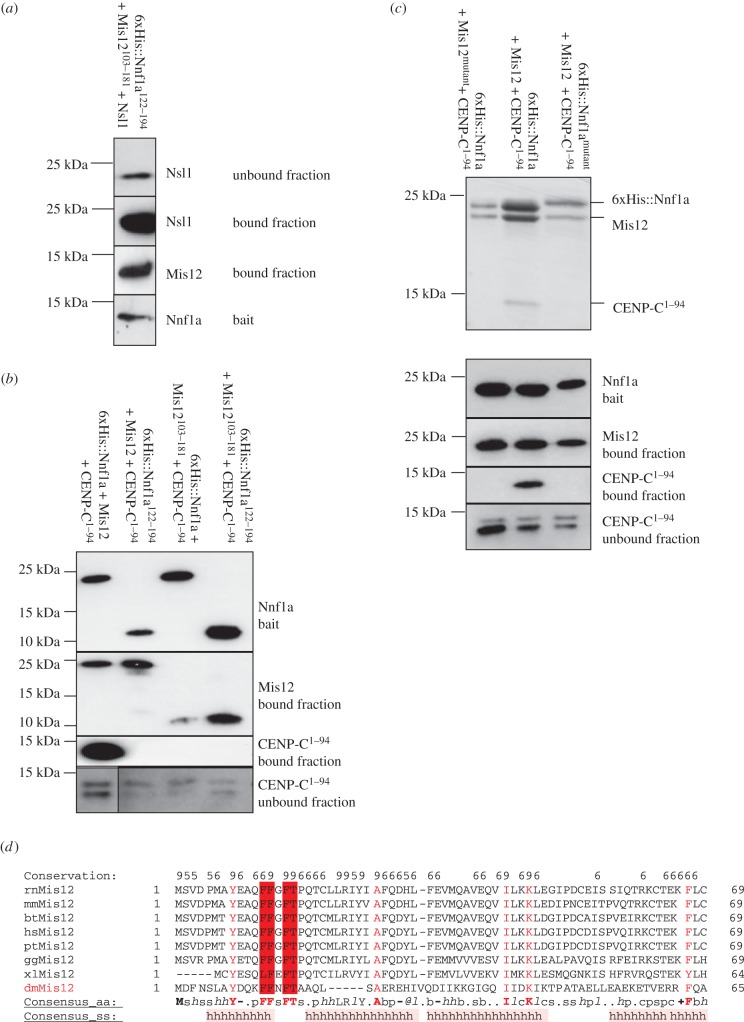
Nnf1a–Mis12 amino-termini form a platform for interaction with CENP-C^1–94^ and their carboxy-termini for interaction with Nsl1. (*a*) WB showing Mis12^103–181^ and Nsl1 co-purified with 6xHis::Nnf1a^122–194^ when co-expressed in *E. coli.* (*b*) WB showing results of *E. coli* co-expressions and co-purifications of truncated Nnf1a–Mis12 proteins together with CENP-C^1–94^. Mis12 and CENP-C^1–94^ co-purified with 6xHis::Nnf1a provide positive control. CENP-C^1–94^ was expressed in all tested samples as indicated by specific bands present in unbound fractions. (*c*) Coomassie-stained SDS-PAGE gel (upper panel) showing results of co-expressions and co-purifications of 6xHis::Nnf1a^mutant^ (W41A, I44A, Y45A) with Mis12 and CENP-C^1–94^ or 6xHis::Nnf1a with Mis12^mutant^ (F12A, F13A, F15A, T16A) and CENP-C^1–94^. 6xHis::Nnf1a co-expressed and co-purified with Mis12 and CENP-C^1–94^ is a positive control. Lower panel shows WB analysis of the same samples. CENP-C^1–94^ was expressed in all tested samples as indicated by specific bands present in unbound fractions on WB analysis. The expression and stability of mutated proteins is confirmed by immunoblotting and CBB staining. (*d*) Multiple sequence alignment of Mis12 proteins built with PROMALS 3D software [35]. Amino acids with highest conservation rate are marked with red letters. Four amino acids (_12_FFxFT_16_) that were mutated to alanines to abolish interaction with CENP-C are highlighted in dark red. rn, *Rattus norvegicus*; mm, *Mus musculus*; bt, *Bos taurus*; hs, *Homo sapiens*; pt, *Pan troglodytes*; gg, *Gallus gallus*; xl, *Xenopus laevis*; dm, *D. melanogaster*.

We next wanted to define interaction regions within the purified trimeric complexes and to this end analysed both trimers by HDX-MS. By comparing deuteration patterns for Mis12 and Nnf1a when they were in complex with either Nsl1 or CENP-C^1–94^, we identified differentially protected peptides that might be good candidates for interacting regions (figure 3). When Nnf1a and Mis12 were in complex with Nsl1, we observed significant protection of the carboxy-terminal peptides of Nnf1a, residues 147–177, and Mis12, residues 134–149 (figure 3*a*). The same residues were fully exposed to HDX when Nnf1a and Mis12 were in complex with CENP-C. This strongly suggested that the carboxy-termini of Nnf1a and Mis12 are both involved in the interaction with Nsl1. The protected regions lie just C-terminally to the previously identified dimerization motifs responsible for the formation of the MN heterodimer (figure 1). Subsequent AP-MS experiments with GFP-tagged, truncated Nnf1a and Mis12 proteins confirmed the HDX data (figure 3*b*). Nsl1 peptides were identified in pull downs in which the fragments Nnf1a^122–194^ and Mis12^103–181^ were used as baits. Interestingly, when full-length proteins in which the Mis12–Nnf1a interaction was disrupted by amino acid mutation within either Mis12 or Nnf1a were used as baits, we could not identify Nsl1 peptides by AP-MS analysis (figure 3*b*). These data further confirm our hypothesis that the MN dimer must be formed first for the interaction with Nsl1 to occur. To determine whether the carboxy-terminal fragment of the MN dimer is sufficient for direct interaction with Nsl1, we co-expressed 6xHis::Nnf1a^122–194^ and Mis12^103–181^ with full-length Nsl1 in *E. coli* using the Duet system. Subsequent affinity purification on Ni-NTA resin revealed complex formation of all three co-expressed polypeptides (figure 4*a*). These results prove that the carboxy-terminally located region within the MN dimer supports the interaction with the third Mis12C component, Nsl1.

### 2.4. Nnf1a and Mis12 interact with CENP-C via their amino-termini

We then searched for peptides required for interactions between the Mis12–Nnf1a dimer and CENP-C. AP-MS analysis revealed that Nnf1a^1–150^ and Mis12^1–132^, segments sufficient to form the MN dimer, are also sufficient to pull down endogenous CENP-C from D.mel-2 cell extracts (figure 3*b*), indicating that the amino-terminal parts of both proteins are indispensable for interaction with CENP-C. To determine whether the amino-terminal parts of these proteins are solely responsible for the direct interaction with CENP-C, we co-expressed truncated carboxy-terminal fragments of either Nnf1a or Mis12, or both, with CENP-C^1–94^ in *E. coli*, and checked whether they could be co-purified (figure 4*b*). Although these C-terminal fragments of Nnf1a and Mis12 were able to bind a partner within the MN dimer, neither 6xHis::Nnf1a^122–194^–Mis12 nor 6xHis::Nnf1a–Mis12^103–181^ could form a complex with CENP-C^1–94^. This clearly indicates that the amino-terminal parts of both Nnf1a and Mis12 are required to bind CENP-C. To narrow down the interacting regions within N-terminal parts of Nnf1a and Mis12, we compared the deuteration patterns for both proteins by analysing MN-CC and MN-N complexes. In the case of Nnf1a, we observed a small region in its amino-terminus (41–53 aa) that became more protected in the MN-CC complex (figure 3*a*) than in MN-N. However, we did not observe any significant differences in deuteration patterns of Mis12 protein when bound within the same complex. To determine whether the region of Nnf1a indicated by HDX-MS indeed is responsible for binding CENP-C, we mutated three hydrophobic residues within that part (W41A, I44A, Y45A), stably expressed the mutant protein in D.mel-2 cells as a fusion with GFP and performed AP-MS analysis. Mutated Nnf1a did not pull down CENP-C (figure 3*b*) although it co-purified with Mis12 and Nsl1, as expected, thus confirming the importance of the identified motif (Nnf1a^41–53^) for the binding of Nnf1a to CENP-C.

To map the potential CENP-C-interacting region within the amino-terminal part of Mis12, for which we did not find any HDX protected peptides, we performed multiple sequence alignment searches for amino acids in the amino-terminal part of Mis12 that are conserved among different species (figure 4*d*). We identified four hydrophobic amino acids (F12, F13, F15, T16) that are highly conserved between Mis12 orthologues, mutated all of these to alanine residues and expressed them in D.Mel-2 cells for AP-MS analysis. Similarly to Nnf1a^W41A,I44A,Y45A^, Mis12^F12A,F13A,F15A,T16A^ did not pull down endogenous CENP-C in such experiments (figure 3*b*), while the mutant was still able to interact with endogenous Nnf1a and Nsl1. Thus, we identify this part of Mis12 as indispensable for the interaction between Mis12C and CENP-C. To further confirm that mutations of residues within these regions abolish direct interaction with CENP-C, we co-expressed recombinant 6xHis::Nnf1a^W41A,I44A,Y45A^–Mis12 and 6xHis::Nnf1a–Mis12^F12A,F13A,F15A,T16A^ with CENP-C^1–94^ in *E. coli* followed by affinity purification on Ni-NTA resin. This revealed that neither the Mis12 nor Nnf1a mutant proteins were able to form a stable complex with CENP-C^1–94^ (figure 4*c*). Thus residues W41, I44, Y45 of Nnf1a and residues F12, F13, F15, T16 of Mis12 are essential for the interaction with CENP-C and both regions are necessary for binding. Similar to the interaction with Nsl1, the formation of the MN dimer is a prerequisite for the interaction with CENP-C, although different parts of the dimer are responsible for the binding to Nsl1 or CENP-C.

### 2.5. Nsl1^102–157^ interacts with the MN dimer

**Figure 5.**
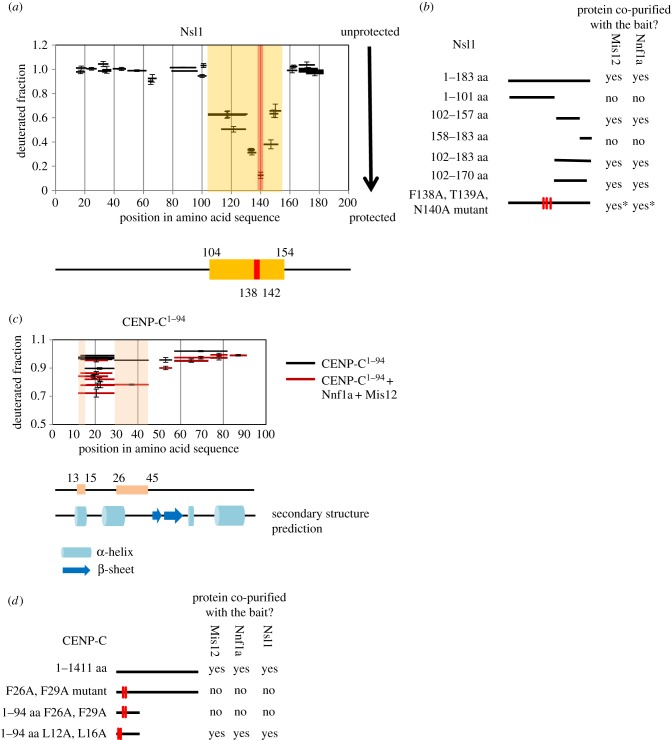
Determining interaction regions in Nsl1 and CENP-C^1–94^ when in complex with Mis12–Nnf1a. (*a*) HDX pattern in Nsl1 when in complex with Mis12–Nnf1a revealing protected (i.e. structured) regions. Graph represents fractional deuteration level of Nsl1 peptides after 10 s of HDX. Black bars represent Nsl1 peptides identified by MS. The only region protected from HDX is indicated by the orange box. The peptide that was the most protected from exchange (^138^FTNAA^142^) is indicated with the red box. Error bars represent standard deviation. (*b*) Schematic of AP-MS results from D.mel-2-cultured cells. Nsl1 truncations were fused with GFP and used as baits. Thick black lines correspond to the length of a particular bait construct. Detailed information about number of proteins' peptides identified by MS and their scores can be found in the electronic supplementary material, table S1. *For this Nsl1 mutant, the number of peptides identified by MS for Mis12 and Nnf1a is significantly lower than in pull down with Nsl1 WT used as bait (see electronic supplementary material, table S1, for details). (*c*) Identification of region in CENP-C^1–94^ showing an increased protection when in complex with Mis12–Nnf1a. Graph represents fractional deuteration level of CENP-C^1–94^ peptides after 10 s of HDX. Horizontal bars represent CENP-C^1–94^ peptides identified by MS. Red bars correspond to CENP-C^1–94^ when it is in complex with Mis12–Nnf1a. Black bars correspond to CENP-C^1–94^ on its own. Pink boxes represent regions of the strongest protection when CENP-C^1–94^ is in complex with MN. Error bars show standard deviation. Secondary structure prediction of CENP-C^1–94^ is aligned to match amino acid sequence on *X*-axis. Blue cylinder corresponds to α-helix, blue arrow to β-sheet. Prediction was made with Phyre2 [36]. (*d*) Schematic of AP-MS results from D.mel-2-cultured cells. CENP-C truncations and mutants were fused with GFP and used as baits. Thick black lines correspond to the length of a particular bait construct. Red marks indicate mutated amino acids. Detailed information about number of peptides identified by MS and their scores can be found in the electronic supplementary material, table S1.

We then decided to narrow down the MN-interacting region within Nsl1 to identify the fragment of this protein that is sufficient for binding to the Mis12–Nnf1a heterodimer. We analysed the MN-N complex by HDX-MS and looked at the deuterium uptake profile of Nsl1 (figure 5*a*). Regions between residues 1–103 and 158–183 were not protected from the H/D exchange and reached maximum deuteration within the first 10 s of incubation in the D_2_O buffer. The only fragment of Nsl1 protein that was at least partly protected from the exchange, and therefore indicating a possible interaction surface, was the region between residues 104 and 154. In order to validate the HDX-MS observations, we created a set of Nsl1 truncations fused with GFP, expressed them in D.mel-2 cells and performed AP-MS analysis. The outcome fully corroborated the HDX-MS findings (figure 5*b*). Constructs encompassing residues 102–157 of Nsl1 were able to pull down both Mis12 and Nnf1a, proving that this region is involved in the interaction with the MN dimer.

Additionally, in our HDX-MS analysis, we identified one peptide that was especially well protected from exchange: ^138^FTNAA^142^. In order to investigate the role of this peptide in the complex formation, we created a full-length Nsl1 construct with three point mutations within the FTNAA peptide. The construct in which the FTN residues were mutated to AAA pulled down Mis12 and Nnf1a proteins but the number of identified Nnf1a and Mis12 peptides was significantly lower than in wild-type (WT) Nsl1 pull down (electronic supplementary material, table S1). This suggests that the short sequence ^138^FTNAA^142^ is important for efficient interaction between Nsl1 and the MN dimer.

### 2.6. CENP-C's interaction with the MN dimer requires two critical phenylalanine residues

To identify the region within CENP-C^1–94^ responsible for its interaction with Mis12 and Nnf1a, we examined the deuteration patterns of CENP-C^1–94^ alone and in complex with MN. When alone, the H/D exchange on CENP-C^1–94^ was very rapid—all of the identified peptides reached maximum deuteration level after only 10 s of incubation in D_2_O buffer (figure 5*c*). This indicates that the analysed fragment is intrinsically unstructured. However, we observed slight protection from exchange in the region covering the first 45 residues of CENP-C^1–94^ when it was in complex with Mis12–Nnf1a (figure 5*c*). The difference in deuterium uptake was not substantial and visible only at the 10 s incubation time point. Longer incubations (1 min, 20 min) resulted in maximum deuteration level of all 94 amino acids (data not shown). This deuteration pattern indicates that the amino acids involved in interaction with Mis12–Nnf1a lie within the first 45 residues of CENP-C and that the interaction itself might not affect the polypeptide backbone but rather the side chains of amino acids present in that fragment.

A secondary structure prediction of CENP-C^1–94^ performed with Phyre2 software indicates that the 45 amino acid N-terminal fragment of CENP-C may form two α-helices. This region overlaps with the part of the protein protected in HDX-MS (figure 5*c*). We therefore introduced point mutations within the motifs covering both α-helices to test whether either of them is involved in the interaction with MN. To do this, we established two D.mel-2 cell lines stably expressing either CENP-C^1–94: L12A,L16A^ (mutations in the first α-helix) or CENP-C^1–94: F26A,F29A^ (mutations in the second *α*-helix) fused to GFP and performed AP-MS analysis to identify interacting partners of the mutant transgenic proteins (figure 5*d*). While CENP-C^1–94: L12A,L16A^ managed to pull down almost all KMN network proteins, just as WT CENP-C^1–94^, we were unable to identify any core kinetochore proteins when CENP-C^1–94: F26A,F29A^ was used as bait. Thus, residues F26 and F29 are required for the interaction with the Mis12C. This suggested that it is not the first short predicted *α*-helix but rather the second longer one which participates in the direct interaction with the MN dimer of the Mis12C. We further confirmed the above results by repeating AP-MS experiments using GFP-tagged full-length CENP-C^WT^ or CENP-C^F26A,F29A^ stably expressed in cultured D.mel-2 cells (figure 5*d*).

**Figure 6.**
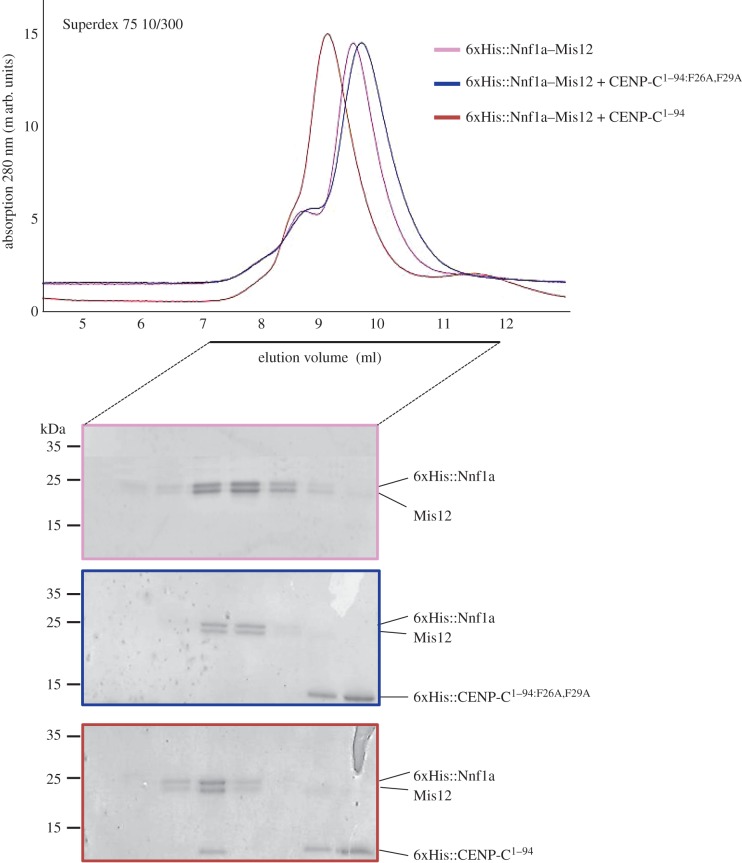
CENP-C^1–94:F26A,F29A^ does not form a complex with Nnf1a–Mis12. SEC runs of recombinant complexes with corresponding Coomassie-stained SDS-PAGE of SEC fractions. 6xHis::Nnf1a was co-expressed and co-purified with Mis12 using *E. coli* pDuet system. 6xHis::CENP-C^1–94:F26A/F29A^ and 6xHis::CENP-C^1–94^ were expressed in *E. coli*, purified separately and added to Mis12–Nnf1a complex prior to SEC run. CENP-C fragment does not absorb UV light at 280 nm, hence no peaks corresponding to either 6xHis::CENP-C^1–94^ or 6xHis::CENP-C^1–94:F26A/F29A^ are visible on chromatograms. 6xHis::CENP-C^1–94:F26A/F29A^ was expressed and stable as indicated by single band visible on Coomassie-stained gel.

To further test the role of these two phenylalanine residues in direct interaction with the MN dimer, we attempted to reconstitute the MN–CENP-C^1–94: (WT versus F26A,F29A)^ complex *in vitro*. To do this, we expressed either 6xHis::CENP-C^1–94^ or 6xHis::CENP-C^1–94: F26A,F29A^ recombinant proteins in *E. coli* and affinity purified them to homogeneity. Both proteins were then mixed with purified 6xHis::Nnf1a–Mis12 heterodimer, respectively, followed by SEC on a Superdex 75 column. We found that while 6xHis::CENP-C^1–94^ co-eluted as a complex with the MN dimer, the 6xHis::CENP-C^1–94: F26A,F29A^ mutant protein could not (figure 6). This further supports the importance of CENP-C residues F26 and F29 for the interaction with the Mis12C.

### 2.7. Physiological consequences of disrupting interaction surfaces within the Mis12 complex

**Figure 7.**
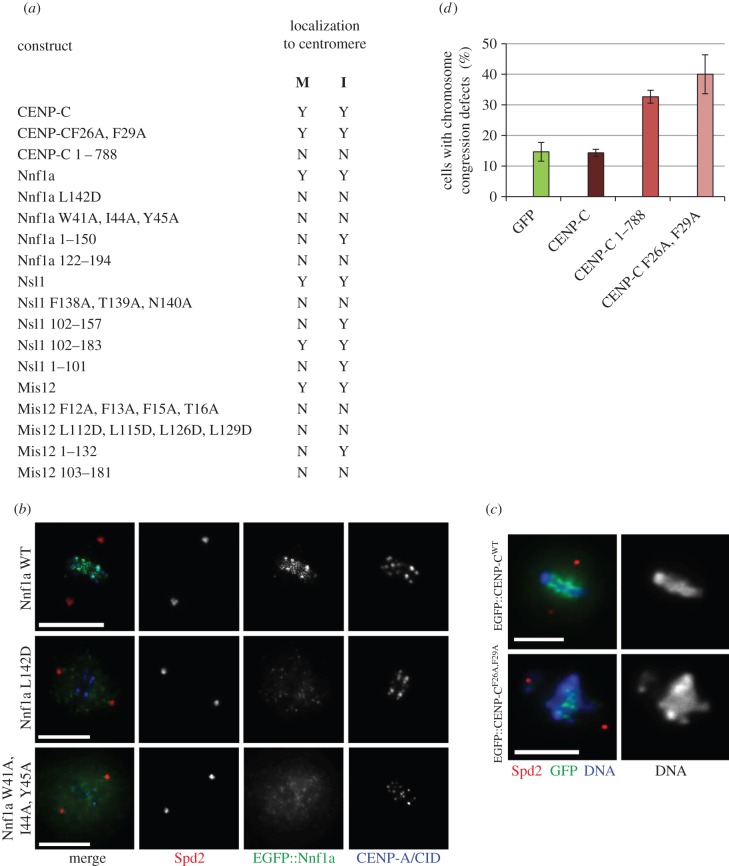
Disruption of interaction surfaces leads to mislocalization of Mis12C components and chromosome segregation defects. (*a*) Table showing localization of different CENP-C, Nnf1a, Mis12 and Nsl1 truncations and mutants in mitosis and interphase of D.mel-2 cells. ‘M’ describes the localization during mitosis, ‘I’ the localization in interphase. (*b*) Mutation of residues responsible for the physical interactions between the Mis12 complex subunits leads to the mislocalization of the kinetochore components. An example is given for cells expressing WT, L142D or a triple W41A, I44A, Y45A mutant of Nnf1a fused with EGFP. Wild-type protein localizes to mitotic centromeres marked by the CID/CENP-A signals, whereas EGFP::Nnf1a^L142D^ and EGFP::Nnf1a^W41A,I44A,Y45A^ are diffuse. Anti-Spd2 staining shows centrosomes. Scale bar in (*c*,*d*) represents 5 µm. (*c*) Expression of the CENP-C^F26A,F29A^ in D.mel-2 cells induces chromosomal aberration phenotype. Examples of control metaphase cells expressing a control EGFP::CENP-C^WT^ construct and a mutant EGFP::CENP-C^F26A,F29A^. Cells were stained with anti-Spd2 to show centrosomes and DAPI to visualize DNA. The graph displaying the quantification of this phenotype is given in panel (*d*). (*d*) Quantification of improperly congressed or scattered chromosomes in EGFP-, EGFP::CENP-C-, EGFP::CENP-C^1–788^- and EGFP::CENP-C^F26A,F29A^-expressing cells. Fourteen per cent of the EGFP-expressing cells (*n* = 100), 14% of the EGFP::CENP-C-expressing cells (*n* = 100), 32% of the EGFP::CENP-C^1–788^-expressing cells (*n* = 100) and 40% of EGFP::CENP-C^F26A,F29A^-expressing cells (*n* = 100) showed chromosome congression defects.

To assess the significance of mutations of newly identified interacting surfaces within the Mis12C, we expressed GFP-tagged mutant or truncated forms of Mis12C components in cultured D.mel-2 cells and observed protein localization by fluorescence microscopy (summarized in figure 7*a*). We found that Nnf1a^W41A,I44A,Y45A^ and Nnf1a^L142D^ did not localize to centromeres either in interphase or mitosis, reflecting their failure to form complexes with the centromeric CENP-C or Mis12, respectively (example in figure 7*b*). Interestingly, Nnf1a^1–150^, the fragment that interacts with Mis12 and CENP-C, localized to centromeres in interphase but not in mitosis. This confirms our earlier observation of the requirement of Nsl1 during the formation of mitotic interactions in the *Drosophila* Mis12C [20,25,26] (Nnf1a^1–150^ does not interact with Nsl1, figure 3*b*). Similar results were observed in the case of Mis12; both multiple amino acid-substitution mutants (Mis12^F12A,F13A,F15A,T16A^ and Mis12^L113D,L115D,L126D,L129D^) failed to localize to centromeres throughout the cell cycle, while the truncated Mis12 fragment, Mis12^1–132^, which interacts with Nnf1a and CENP-C, localized to the centromere exclusively in interphase (due to the impaired interaction with Nsl1). These results also show that the N-terminal regions of Nnf1a and Mis12 together comprise a centromere localization domain that places Mis12C in the centromeric region shortly before mitosis and enables further attachment of outer kinetochore proteins.

Introduction of mutations into the amino-terminal part of CENP-C did not interfere with its localization, because the centromere-binding domain of CENP-C is situated in its C-terminal part [37,38]. In agreement with this, another truncated CENP-C protein, CENP-C^1–788^, was unable to localize to centromeres (figure 7*a*), as predicted [14]. Since our data showed that CENP-C^F26A,F29A^ cannot interact with the Mis12C, we wanted to test whether the presence of the mutated protein triggers formation of improper chromosome attachments leading to chromosome segregation defects. We examined mitotic cells expressing GFP::CENP-C^F26A,F29A^, GFP::CENP-C, GFP::CENP-C^1–788^ or GFP alone and determined whether chromosomes were properly aligned at the metaphase plate. Consistent with the previously published results [14], we found a significant increase in the proportion of cells with misaligned chromosomes and congression defects when cells expressed either GFP::CENP-C^1–788^ or GFP::CENP-C^F26A,F29A^ (example shown in figure 7*c*, quantified in figure 7*d*). This further confirms the results obtained from *in vitro* binding experiments and underscores the importance of CENP-C residues F26 and F29 for the interaction with the Mis12C and the process of kinetochore assembly in *D. melanogaster*.

## 3. Discussion

Kinetochore proteins are recruited to centromeres shortly before each cell division to form functional complexes [2]. How exactly kinetochore assembly is regulated is largely unknown. In *Drosophila* embryos, the Mis12 complex and Spc105/Knl1 are the first KMN components to be assembled [25]. Super-resolution microscopy has shown the Mis12C components to be localized closest to mitotic centromeres [25], and mutations in the Mis12C subunits Mis12 and Nsl1 lead to similar chromosome segregation defects [26]. Consistently, in other species, Mis12C was also found to be at the foundation of the kinetochore [22,39–41]. Together, this points to the Mis12C being key for formation of the KMN network and binding of other auxiliary proteins (reviewed in [2,8]). Our previous studies revealed that *Drosophila* CENP-C is essential and sufficient for kinetochore formation and that its amino-terminal fragment is responsible for the kinetochore assembly [14]. CENP-C is also subjected to multiple post-translational modifications, including phosphorylation, suggesting that this protein may serve as a regulatory hub for the kinetochore assembly [42]. Here, we have addressed how CENP-C interacts with the subunits of the Mis12C to form an interface between the mitotic centromere and kinetochore. To do so, we mapped the direct interacting surfaces between CENP-C and the *Drosophila* Mis12C components.

The *Drosophila* Mis12C comprises Mis12, Nnf1 and Nsl1 and appears to be missing the Dsn1 subunit seen in vertebrates. We knew from proteomic studies that the two paralogues of Nnf1, Nnf1a and Nnf1b [20,21], form separate assemblies in *Drosophila* such that in any given *Drosophila* Mis12 complex, either Nnf1a or Nnf1b is present but never both (data not shown). Therefore, in this study, we studied complex formation with just one paralogue, Nnf1a, assuming that its binding mode is similar to Nnf1b (multiple sequence alignments show high level of conservation in key residues involved in binding to Mis12 and CENP-C—data not shown), although the developmental pattern of expression of those two isoforms suggests age-related regulation and hence the possibility of some differences [21].

**Figure 8.**
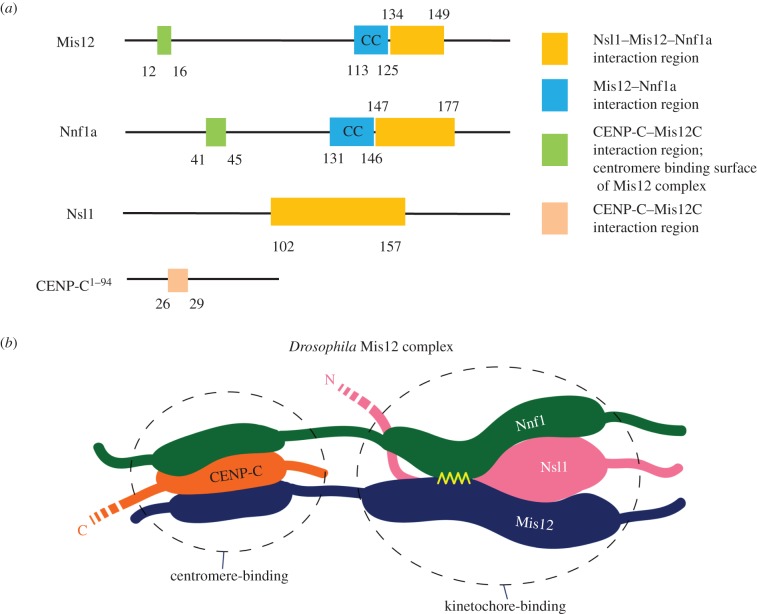
Mis12–Nnf1a form a platform for interaction with CENP-C and Nsl1. (*a*) Schematic of interaction regions in CENP-C, Nnf1a, Mis12 and Nsl1. Coloured boxes show protein regions that were identified as interacting surfaces in this study. (*b*) Cartoon showing the hypothetical organization of the *Drosophila* Mis12 complex emerging from this study. Amino-terminal ends of the MN dimer (the centromere-binding part) are anchored at the centromeric chromatin via CENP-C. Carboxy-terminal fragments of the MN dimer (the kinetochore-binding part) serve as scaffold for binding outer kinetochore components, including Spc105/KNL1 and the Ndc80 complex. The yellow ribbon represents the CC interaction between Mis12 and Nnf1.

We used HDX-MS to identify regions protected from the rapid H/D exchange as potentially indicating surfaces of direct protein–protein interactions as well as areas buried within individual subunits or allosteric interactions between distant protein regions. Moreover, HDX probes only the protection of amide hydrogens and not interactions of amino acid side chains. In order to discriminate between the intra- and intermolecular interactions predicted by HDX, we therefore applied additional mutational analysis, AP-MS and microscopy. Thus from our combined data, we could identify domains of the Mis12C components and CENP-C involved in direct protein–protein interactions (summarized in figure 8*a*).

We found that Mis12 and Nnf1a form a stable heterodimer *in vitro*. The previous literature on yeast and human Mis12 complexes had provided clues that Mis12 and Nnf1a interact directly [23,29,30,33]. Our study not only confirms this interaction but goes further by showing that the formation of a dimer (that we term MN) is absolutely necessary for binding either centromeric CENP-C or the other Mis12C component, Nsl1. Mis12 and Nnf1a appear to bind each other via a CC structure that resembles a classical leucine zipper. There is one such domain located within the carboxy-terminal parts of each of those two proteins (figure 8*a*). Our results indicate that the dimer has an extended shape suggesting that the Mis12 and Nnf1a polypeptides may assume a parallel conformation with their amino-termini binding the centromere and carboxy-termini pointing towards the outer kinetochore. Although the rod-like shape of the entire Mis12C has been reported previously by others [23,28,29], here we propose that the shape of the entire Mis12C is in fact imposed by the properties of the MN dimer.

Adjacent to the Mis12–Nnf1a dimerization domain, or perhaps even overlapping with it, lies a region responsible for interaction with Nsl1. It is possible that Nsl1 binds to that part of the MN dimer forming a triple CC, although the predictions for the MN-interacting part of Nsl1 do not show any high probability for CC formation in that region of the protein (data not shown). HDX-MS data clearly show that Nsl1 uses its carboxy-terminal part to bind the MN dimer. It is not yet clear what exactly the amino-terminal part of Nsl1 interacts with. The function of the N-terminus of human Nsl1 also remains unknown, although a recent structural study suggests that it meanders along the length of the entire Mis12C, perhaps even reaching the kinetochore–centromere interface [33].

Targeted amino acid substitutions introduced to the amino-terminal regions of either Mis12 or Nnf1a disrupt their interaction with the centromere, because those N-terminal parts of the MN dimer directly bind CENP-C (figure 8*a*, green areas). Consequently, point mutations within these parts of Mis12 or Nnf1a prevent the proteins from localizing to centromeres. However, the same mutations still permit formation of an entire trimeric *Drosophila* Mis12 complex comprising Mis12, Nnf1 and Nsl1 (figure 3*b*). This is in agreement with our previous observations that the entire KMN network can be recovered from *Drosophila* cells, unbound to CENP-C [20]. Proteomic studies suggest that the *Drosophila* KMN network is itself a stable structure, which most likely does not require any mitosis-specific conformational changes or post-translational modifications in order to be assembled de novo. This leads to the notion that regulation of pre-mitotic kinetochore assembly is either through accessibility of kinetochore components (as suggested previously [25,43]) or through modification of the interface between centromeres and kinetochores (CENP-C), or both.

In *Drosophila*, just the single CENP-C-dependent kinetochore assembly pathway seems to be active, in contrast to vertebrates where the CENP-T/W pathway is also operational (reviewed in [2]). It is therefore astounding that a short stretch of amino-terminal end of CENP-C can be sufficient to support the entire interaction network between chromosomes and MT fibres of the mitotic spindle. It will be important to clarify whether protein–protein associations between CENP-C and the Mis12 complex, which we and others recently described [14,15], are indeed solely responsible for the dynamic binding between chromosomes and MTs or if there are other components and interactions yet to be identified that can contribute to regulation of the metazoan kinetochore assembly.

The data presented here point to the importance of studying reconstituted recombinant multiprotein assemblies to attain better understanding of protein–protein interactions. In the case of the *Drosophila* Mis12 complex, two-hybrid screens revealed only two subunits of the complex interacting directly. In fact, all other interactions require more than two components and therefore could not be detected by Y2H. However, reconstitution experiments must be followed up by additional approaches to validate the results of *in vitro* binding. In this study, we used a combination of cellular proteomics, mutagenesis and microscopy to evaluate HDX-MS data. Together, they led to the conclusion that Mis12 and Nnf1a proteins adopt a parallel configuration to form an elongated dimer through a leucine zipper between their carboxy-terminally located CCs (figure 8*b*). That region of the MN dimer is also responsible for binding Nsl1 protein, which uses its C-terminal part to bind the Mis12 and Nnf1a dimer. The amino-terminal ends of Mis12 and Nnf1a, on the other hand, form a centromere-binding surface and directly associate with CENP-C's N-terminus (figure 8*b*). Our results indicate that indeed the Mis12 complex assumes an extended conformation, but it is rather due to the extended structure of the MN dimer, not because the components of the complex bind consecutively one after another. It will now be interesting to determine the precise position of Spc105/KNl1 in relation to its role in the formation of *Drosophila* Mis12 complex. Moreover, it will be of great interest to known whether the same features of the molecular interactions in the trimeric Mis12 complex of *Drosophila* relate to the intermolecular interfaces existing within the tetrameric human Mis12 complex.

## 4. Material and methods

### 4.1. Plasmids

A detailed list of expression constructs and recombinant plasmids is available in the electronic supplementary material, table S3. For Y2H and Duet vectors, protein-coding sequences were subcloned into DNA vectors using conventional cloning. All the expression vectors used for AP-MS studies were generated with the Gateway technology (Invitrogen). pMT-GFP and pMT-DESTNPta vectors used for generation of expression vectors were previously described [44,45]. Premature STOP codons in truncated constructs were introduced by site-directed mutagenesis using appropriate PCR primers. QuickChange Mutagenesis Kit (Stratagen) was used to introduce all amino acid-substitution mutations. All constructs were verified by DNA sequencing.

### 4.2. Protein expression and purification

Recombinant proteins were expressed in *E. coli* strain BL21(DE3) (Life Technologies) following standard procedures. Briefly, bacteria were transformed or co-transformed with recombinant Duet plasmids encoding the desired proteins (electronic supplementary material, table S3) and grown at 37°C to A600 ∼ 0.7 in Luria Broth supplemented with standard concentration of the appropriate antibiotics. Protein expression was induced with 0.5 mM isopropyl-β-d-1-thiogalactopyranoside (IPTG) at 21°C for 3 h, with the exception of 6xHis::Nsl1 + Nnf1a + Mis12, which was induced overnight. Bacterial cells were harvested, resuspended in buffer A (20 mM Tris/HCl pH 8.0, 300 mM NaCl, 10% glycerol, 2 mM 2-mercaptoethanol, 20 mM imidazole) supplemented with EDTA-free protease inhibitor cocktail (Roche) and 1 mg ml^−1^ lysozyme (Sigma Aldrich) and incubated on ice for 30 min. Cells were lysed by sonication and clarified by centrifugation at 14 000*g* for 20 min at 4°C. The cleared lysates were incubated with Ni-NTA agarose beads (QIAGEN) for 1 h at 4°C. Beads with bound proteins were consecutively washed with 50 volumes of buffer A supplemented with 20 mM and 30 mM imidazole. Bound proteins were eluted with buffer A supplemented with 230 mM imidazole. Eluates were then subjected to SEC.

### 4.3. SEC and MALS-SEC

For SEC, we used Superdex 200 10/300 (GE Healthcare) or Superdex 75 10/300 (GE Healthcare) columns pre-equilibrated with buffer containing 300 mM NaCl, 20 mM Tris–HCl pH 8.0, 10% (vol/vol) glycerol and 2 mM 2-mercaptoethanol. Ni-NTA-purified protein samples were loaded onto the columns and SEC was run at 0.5 ml min^−1^ flow rate at room temperature. Elution of proteins was monitored at 280 nm. Fractions were collected and analysed by SDS-PAGE and Coomassie Brilliant Blue (CBB) staining. For HDX-MS studies, main fractions, with the highest protein content, were used. SEC combined with MALS analysis was performed using an in-line MALS detector (DAWN HELEOS–II, Wyatt Technology) and differential refractometer (Optilab T-rEX, Wyatt Technology). Samples were run at 0.5 ml min^−1^ flow rate at 4°C on a Superdex 200 10/300 (GE Healthcare) column, equilibrated with MALS-SEC buffer (150 mM NaCl, 10 mM Tris/HCl pH 8.0). Results were analysed using Astra v. 6 software according to the manufacturer's instructions.

### 4.4. *In vitro* complex formation

To analyse formation of distinct complexes, 6xHis::CENP-C^1–94:F26A,F29A^, 6xHis::CENP-C^1–94^ and 6xHis::Nnf1a–Mis12 dimer were expressed in *E. coli*, and purified as described in the ‘Protein expression and purification’ section. 6xHis::Nnf1a–Mis12 dimer was then mixed with either 6xHis::CENP-C^1–94:F26A,F29A^ or 6xHis:CENP-C^1–94^, incubated for 30 min on ice and then loaded on a Superdex 75 column. SEC was run and fractions were collected and analysed by SDS-PAGE and CBB staining.

### 4.5. Duet system interaction experiments

Protein–protein interactions and complex formation were analysed *in vitro* by co-expressing recombinant proteins in *E. coli* exploiting the advantages of the Duet vector system (Merck Millipore). Proteins were co-expressed and co-purified as described in ‘Protein expression and purification’. Eluates were subjected to 15% SDS-PAGE. Presence of bait, binding partners and expressed proteins that did not bind to the bait was visualized by western blotting (WB) and/or CBB staining. After CBB staining, protein bands were cut out and analysed by mass spectrometry to further confirm the proteins' presence. The following primary antibodies were used in WB experiments: rabbit anti-Mis12 (1 : 2000), sheep anti-Nnf1a (1 : 2000), rabbit anti-Nsl1 (1 : 2000) (all have been generated and used previously [14,25,26]) and mouse anti-CENP-C^1–188^ (1 : 1000). The latter was generated in our laboratory as described below. Secondary antibodies were obtained from Jackson ImmunoResearch (horseradish peroxidase conjugates) and used at 1 : 1000 dilution.

### 4.6. Antigen preparation for immunization

His::CENP-C^1–188^ was expressed in *E. coli* strain BL21(DE3) (Life Technologies) and purified to homogeneity as follows: cells were grown to OD_600_ = 0.5 in 2 × 800 ml LB and expression was induced with 0.4 mM IPTG for 3 h at 37°C. Cells were lysed by sonication in buffer containing 50 mM NaH_2_PO_4_ pH 8.0, 300 mM NaCl, 10 mM imidazole, 5 mM 2-mercaptoethanol, 1 mM phenylmethanesulfonyl fluoride and complete protease inhibitor cocktail (Roche, 11873580001) supplemented with 1 mg ml^−1^ lysozyme (Sigma Aldrich) and centrifuged for 20 min at 4°C at 34 000*g* to pellet cell debris. Inclusion body of His::CENP-C^1–188^ was resuspended in denaturing buffer (DB) containing 20 mM Tris pH 8.0, 500 mM NaCl, 20 mM imidazole, 5 mM 2-mercaptoethanol and 6 M guanidine hydrochloride, mixed for 30 min at room temperature followed by centrifugation for 20 min at 4°C at 34 000*g* to remove insoluble debris. From the supernatant, His::CENP-C^1–188^ was purified on Ni-NTA agarose (Qiagen) beads under denaturing conditions and refolded by following standard procedures: beads were gradually washed in DB (without guanidine hydrochloride) supplemented with 6, 4, 2, 1 or 0 M urea, respectively. Antigen was eluted from the beads with buffer containing 20 mM Tris pH 8.0, 500 mM NaCl, 300 mM imidazole and 5 mM 2-mercaptoethanol, concentrated on an Amicon Ultra centrifugal filter (Millipore) and further purified by SEC (Superdex 75, GE Healthcare) equilibrated in PBS. The purity of the samples was analysed by SDS-PAGE, then main fractions were combined, concentrated and used as antigen (100 µg boost^−1^) to immunize mice (Harlan, UK). The specificity of the antibody was confirmed by immunoblotting after gene-specific RNAi in D.Mel-2 cells (electronic supplementary material, figure S6).

### 4.7. Yeast two-hybrid assay

Protein–protein interactions were analysed by the Matchmaker Gold Two-Hybrid system using yeast strains Y182 and Y2H Gold (Clontech Laboratories) transformed with either pGAD424 or pGBT9 constructs encoding the desired proteins. Transformations were performed according to the manufacturer's guide. After transformation, the strains were mated and diploids were analysed for growth on media selecting for the activation of different reporter genes. Diploids were first plated on SD/-Leu/-Trp/X-*α*-Gal plates. The growth was tested after 3 days. The combinations that gave positive result were tested using more stringent conditions (SD/-His/-Leu/-Trp//X-*α*-Gal and SD/-Ade/-His/-Leu/-Trp/X-*α*-Gal plates). All combinations that gave positive results in the lowest stringency conditions were also positive in higher stringency conditions. For CENP-C^1–788^ (cloned into pGBKT7 vector) and Nnf1a, Mis12 mutants forming protein–protein interactions were verified by co-transforming Y2H Gold strain. Co-transformation was performed according to standard procedures. Transformed cells were plated on SD/-Leu/-Trp, SD/-Ade/-Leu/-Trp and SD/-Leu/-Tpr/+aureobasidin selection plates. Combinations that were able to growth on all selection media were considered positive interactions. For positive control experiments, plasmid encoding the SV40 T-antigen fused to the GAL4 activation domain (GAL4 AD) and p53 fused to the GAL4 DNA-binding domain (GAL4 BD) were used. For negative control, empty pGAD424 and pGBT9 vectors were used. To test for auto-activation pGAD424 vectors containing GAL4 AD-fused proteins and pGBT9 vectors containing GAL4 BD-fused proteins were tested against empty pGBT9 and pGAD424 vectors, respectively. The results of Y2H experiments are shown in the electronic supplementary material, table S2.

### 4.8. Cell culture

D.mel-2 cells (Invitrogen) were grown in Express Five SFM (Invitrogen) media according to standard procedures. Stable cell lines were made as described previously [44,45] using FuGENE HD (Roche) transfection reagent. Protein expression was induced by adding copper sulfate to cell culture media (final concentration 0.5 mM, overnight induction). For the purpose of imaging, cells were transferred onto glass coverslips, fixed with formaldehyde and stained as described in Immunofluorescence and microscopy.

### 4.9. Affinity purifications from cultured cells followed by mass spectrometry (AP-MS)

Detailed protocols for making IgG beads and for the protein A affinity purification from Dmel-2 cells were published previously [44]. For the affinity purifications of GFP-fusion proteins [45], we lysed approximately 10^9^ D.Mel-2 cells in 10 ml of lysis buffer (LB; 10 mM Tris pH 7.5, 200 mM NaCl, 0.5 mM EGTA, 0.5% NP-40, 1 mM DTT, 5% glycerol and complete protease inhibitor cocktail) on ice with a PowerGen 125 homogenizer (Fisher Scientific). Homogenates were treated with 2000 Kunitz units of DNase I (Sigma, D4263) for 10 min at 37°C and 10 min at room temperature and centrifuged (4°C, 10 min, 12 100*g*). Clarified lysates were mixed with GFP-Trap agarose beads (Chromotek) for 2 h at 4°C. Beads were washed five times in LB and stored in minimal volume of LB at 4°C prior to mass spectrometry analysis.

The results of AP-MS experiments relevant for this study are shown in the electronic supplementary material, table S1. The values of Mascot scores for individual proteins (‘Protein score’) and number of identified peptides (‘Matches’) were analysed as a measure of ability for certain baits to co-purify with the components of the Mis12C or CENP-C.

### 4.10. Immunofluorescence

For immunofluorescence experiments, cells were harvested, seeded onto coverslips and allowed to adhere for 3 h before fixation. Cells were fixed with 4% formaldehyde (diluted in PBS) for 12 min. Next, they were incubated for 1–3 h in blocking solution (3% BSA, 0.5% Triton X-100 in PBS) and stained for 3 h with primary antibodies diluted in PBT (1% BSA, 0.1% Triton X-100 in PBS). After three washes with PBT, secondary antibody staining was performed for 1 h, followed by another three washes with PBT and one wash with PBS. Coverslips were mounted on slides with ProLong Gold antifade reagent (Invitrogen) with DAPI (for co-localization studies) or without DAPI (for chromosomal aberration phenotype scoring). For co-localization studies, the following primary antibodies were used: chicken anti-CID/CENP-A (1 : 2000), rabbit anti-Spd2 (1 : 2000) and mouse anti-GFP (Roche, 11814460001; 1 : 1000), used as described previously [14]. For chromosomal aberration phenotype scoring, we used chicken α-dPlp [46] (1 : 1000), rabbit anti-phospho histone H3 (Millipore; 1 : 500) and mouse anti-GFP (Roche, 11814460001; 1 : 1000). Secondary antibodies conjugated with AlexaFluor dyes (488 for green, 647 for far-red, 405 for blue or 568 for red channel; Invitrogen) were diluted 1 : 500. Microscopy was performed using a Zeiss Axiovert 200M microscope (100× objective) equipped with a Photometrics CoolSNAP HQ2 camera.

### 4.11. Phenotypic analysis

The chromosomal aberration phenotype was most reliably scored in cells in which the spindle axis was parallel to the coverslip with well-separated centrosomes, as shown by dPLp staining. A minimum of 100 mitotic cells were scored for each variant within each experiment. Each experiment was repeated three times. Only green cells but not those with very high levels of overexpression of GFP-fusion transgenes were scored. Obvious and apparent misaligned chromosomes during metaphase were counted as ‘congression defects’ (figure 7*d*). This stringent classification of phenotypes most likely leads to the underestimation of the real total number of chromosomal aberrations.

### 4.12. Nuclear magnetic resonance

2D-TOCSY and 2D-ROESY spectra were recorded for 100 µM samples of Nnf1a^122–147^ and Mis12^101–130^, and for a 1 : 1 mixture of 200 µM samples of both peptides, on Agilent NMR 600 MHz spectrometer at 283 K. All spectra were processed with the aid of NMRPipe [47] and further analysed using the Sparky v. 3 [48] program. Due to severe limitation in the peptides' solubility, only sequential (i.e. *i*, *i* + 1) NOEs were observed in ROESY spectra. However, due to the sequence heterogeneity, detailed analysis of the spectra enabled resonances to be assigned unequivocally for the methyl groups of L124, V128, L135, A136, A139, L142, I144, A145, A147 of Nnf1a^122–147^ and T101, T109, A110, L112, L115, A117, A124, L126, A127, L129 of Mis12^101–130^. The 2D-TOCSY spectrum recorded for the mixture of peptides showed variations in the location of resonances assigned to residues ^130^F-I^144^ of Nnf1a^122–147^ and ^109^T-L^126^ of Mis12^101–130^, thus identifying the putative dimerization interface.

The NMR data were acquired at the laboratory of Professor Wiktor Kozminski (CENT3, University of Warsaw, Poland).

### 4.13. Molecular modeling

Analysis of the putative structure of Nnf1a–Mis12 has been done with the aid of the YASARA package [49] using crystal structure of the CC domain of *C. elegans* SAS-6 (pdb4gkw) [50] as a template. Nnf1a^115–196^ and Mis12^92–177^ were iteratively aligned with the template molecules using 60 register shifts for each of them with no gaps permitted. The resulting 3600 models were then scored according to the number of leucine side chains forming leucine zipper (see additional data in electronic supplementary material, figure S6, and also its legend) and further compared with the NMR-derived interface (^130^F-I^144^ of Nnf1a^122–147^ and ^109^T-L^126^ of Mis12^101–130^).

### 4.14. Circular dichroism

Peptides Mis12 T_101_SEEEEQKTARLEELKAKYRENMAMLAHLK_130_ and Nnf1a R_122_FLDFSVEFMEQQLASQAKELEIAMA_147_ with C-termini amidated and the N-termini acetylated were purchased from GenScript (Piscataway, NJ).

Dimer reconstitution: 2 mg of Mis12 peptide were dissolved in 0.1 M PBS, pH 7.4. Peptide Nnf1 was dissolved in 0.1 M PBS, pH 10.1, and pH was subsequently shifted to 7.4 with HCl. The concentration of stock solutions was measured using DirectDetect (Millipore) and verified using a spectrophotometer by collecting UV absorption spectra at 220–300 nm and assuming the extinction coefficients for Mis12 peptide 1490 M^−1^ cm^−1^ at 275 nm (1 Tyr residue in the sequence) and for Nnf1 585 M^−1^ cm^−1^ at 257 nm (3× Phe residues in the sequence).

CD spectra of dilution series of homomeric preparations of peptides and the equimolar mixtures of the peptides were carried out using a Jasco J-815 CD spectrometer in a 1 mm quartz cuvette, and the spectra were collected in the range 195–270 nm, at room temperature.

### 4.15. Mass spectrometry

Peptides mixtures were analysed by LC-MS-MS/MS (liquid chromatography coupled to tandem mass spectrometry) using the Nano-Acquity (Waters) LC system and Orbitrap Velos mass spectrometer (Thermo Electron Corp., San Jose, CA, USA). Prior to the analysis, proteins were subjected to standard ‘in-solution digestion’ procedure during which proteins were reduced with 100 mM DTT (for 30 min at 56°C), alkylated with 0.5 M iodoacetamide (45 min in darkroom at room temperature) and digested overnight with trypsin (sequencing Grade Modified Trypsin, Promega V5111). Peptide mixture was applied to an RP-18 precolumn (nanoACQUITY Symmetry^®^ C18, Waters 186003514) using water containing 0.1% TFA as mobile phase and then transferred to a nano-HPLC RP-18 column (nanoACQUITY BEH C18, Waters 186003545) using an acetonitrile gradient (0–60% AcN in 120 min) in the presence of 0.05% formic acid with a flowrate of 150 nl min^−1^. The column outlet was directly coupled to the ion source of the spectrometer working in the regime of data-dependent MS to MS/MS switch. A blank run ensuring lack of cross contamination from previous samples preceded each analysis. Acquired raw data were processed by Mascot Distiller followed by Mascot Search (Matrix Science, London, on-site licence) against FlyBase (in the case of affinity purifications from *D. melanogaster* cell extracts) or NCBI (in the case of Duet co-purifications from *E. coli* cell cultures) databases. Search parameters for precursor and product ion mass tolerance were 20 ppm and 0.6 Da, respectively, with search parameters set as follows: one missed semitrypsin cleavage site allowed, fixed modification of cysteine by carbamidomethylation and variable modification of lysine carbamidomethylation and methionine oxidation. Peptides with Mascot score exceeding the threshold value corresponding to less than 5% false positive rate, calculated by Mascot, were considered to be positively identified.

### 4.16. Hydrogen–deuterium exchange mass spectrometry (HDX-MS)

The list of peptic CENP-C^1–94^, Nnf1a, Mis12, Nsl1 peptides was established using non-deuterated protein samples. For this aim, a 5 µl aliquot of protein sample (at least 25 µM in 300 mM NaCl, 20 mM Tris–HCl pH 8.0, 10% (vol/vol) glycerol and 2 mM 2-mercaptoethanol) was diluted 10 times by adding 45 µl of 20 mM Tris pH 8 and 150 mM NaCl (H_2_O Reaction buffer). Next, the sample was acidified by mixing with 10 µl of 2 M glycine buffer, pH 2.5 (H_2_O Stop buffer) and injected into the nanoAQUITY UPLC system (Waters, Milford, MA, USA). The sample was digested online using an immobilized pepsin column (Porozyme, ABI, Foster City, CA, USA) with 0.07% formic acid in water as mobile phase (flow rate 200 µl min^−1^). Digested peptides were next trapped on a C_18_ column (ACQUITY BEH C18 VanGuard Pre-column, Waters) and then were directed into a reverse phase column (Acquity UPLC BEH C18 column, Waters) with a 6–40% gradient of acetonitrile in 0.1% formic acid at 40 µl min^−1^ using nanoACQUITY Binary Solvent Manager. Total time of a single run was 13.5 min. All fluidics, valves and columns were kept at 0.5°C using HDX Manager (Waters). The pepsin column was kept at 13°C inside the temperature-controlled digestion compartment of the HDX manager. Leucine–enkephalin solution (Sigma) was used as a Lock mass. For protein identification, mass spectra were acquired in MSE mode over the *m*/*z* range of 50–2000. The spectrometer parameters were as follows: ESI positive mode, capillary voltage 3 kV, sampling cone voltage 35 V, extraction cone voltage 3 V, source temperature 80°C, desolvation temperature 175°C and desolvation gas flow 800 l h^−1^. Peptides were identified using ProteinLynx Global Server software (Waters). The list of identified peptides containing peptide *m*/*z*, charge and retention time was further processed with DynamX v. 2.0 program (Waters). Hydrogen–deuterium exchange experiments were carried out as described for non-deuterated samples with the Reaction buffer prepared using D_2_O (99.8% Armar Chemicals, Switzerland), in which the pH_read_ (uncorrected meter reading) was adjusted using DCl or NaOD (Sigma). Five microlitres of protein stock was mixed with 45 µl D_2_O Reaction buffer and exchange reaction was carried out for a specific time period (either 10 s, 1 min or 20 min) at room temperature. The exchange was quenched by reducing the pH_read_ to 2.5 by adding the reaction mixture into an Eppendorf tube containing ice-cold Stop buffer (2 M glycine buffer, pH_read_ 2.5). Immediately after quenching, the sample was manually injected into the nanoACQUITY (Waters) UPLC system. Further pepsin digestion, LC and MS analysis were carried out exactly as described for the non-deuterated sample. To assess minimum exchange (in-exchange control), D_2_O Reaction buffer was added to Stop buffer cooled on ice prior to protein stock addition and the mixture immediately subjected to pepsin digestion and LC-MS analysis as described above. The deuteration level in the in-exchange experiment was calculated using DynamX and denoted as 0% exchange (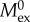). For out-exchange analysis, the 5 µl protein stock was mixed with 45 µl of D_2_O Reaction buffer, incubated overnight in 4°C to avoid protein degradation, mixed with Stop buffer and analysed as described above. The deuteration level in the out-exchange experiment was calculated and denoted as 100% exchange (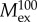). All HDX experiments and control experiments were repeated three times, and the final results represent a mean of these triplicates.

### 4.17. HDX-MS data analysis

The peptic peptide list obtained from the PLGS program was filtered in DynamX v. 2.0 with the following acceptance criteria: minimum intensity threshold, 2000 and minimum products per amino acids, 0.2. The analysis of the isotopic envelopes after exchange was carried out in DynamX v. 2.0 with the following parameters: RT deviation ±15 s, *m*/*z* deviation ±12.5 ppm and drift time deviation ±2 time bins. The average mass of peptides in exchange experiment (*M*_ex_) and the two control experiments (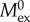 and 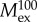) obtained from the automated analysis were then manually verified. Ambiguous or overlapping isotopic envelopes were discarded from further analysis. Final data were exported to an Excel (Microsoft) spreadsheet for calculation of H/D exchange mass shifts and fraction of exchange calculation. For Mis12–Nnf1a–CENP-C^1–94^, Mis12–Nnf1a–Nsl1 and CENP-C^1–94^ samples, the fraction of exchange (*f*) of a given peptide was calculated by taking into account both control values, following the formula:
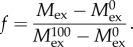


For Mis12–Nnf1a samples, the fraction of exchange (*f*′) of a given peptide was calculated by using a formula which takes into consideration the total number of amino acids in the peptide (aa's), the fast back exchange of hydrogen on the N-terminus (Nterm) and the lack of deuterium exchange in proline residues (Pro):
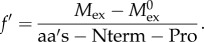


Error bars for exchange fraction (*f* and *f* ′) are calculated as standard deviations of at least three independent experiments. The difference in exchange (ΔHDex) between Mis12–Nnf1a–CENP-C^1–94^and Mis12–Nnf1a–Nsl1 complexes was calculated by subtracting exchange fraction of complex 1 from that of the complex 2. Here, the error was estimated as square root of sum of their variances.

## Supplementary Material

Supplementary Materials
