# Supplementary material for: Network of protein interactions within the Drosophila inner kinetochore
Source: Open Biol. 2016 Feb 24;6(2):150238. doi: 10.1098/rsob.150238 (PMC4772809; doi:10.1098/rsob.150238)
Supplement: Supplementary Materials [file rsob150238supp1.pdf]

Electronic Supplementary Materials

Supplementary Figure S1. Modelling of the coiled-coil based interaction between Mis12 and Nnf1a.

3600 alternative structural alignments of Mis12 and Nnf1a proteins on the reference coiled-coil structure, scored according to the number of intermolecular Leu-Leu contacts. All structures with more than 8 contacts are denoted by circles. The best alignments (difference in register shifts by 14 aa), represented by solid line, identify five highly-scored structures (3 with 13 and 2 with 12 Leu-Leu contacts, respectively), all of which are almost equivalent due to (i,i+7) pseudo-translational symmetry of a coiled-coil, known as heptad repeat [51]. The alternative set of alignments (dotted line, relative shift between registers either 10 or 11aa) identify next four slightly less-scored structures (2 with 12 and 2 with 11 contacts), however, according to Mann's-Whithey U-test, this subsets is scored lower at p = 0.05.

Supplementary Figure S2. Circular dichroism measurements show that Nnf1a$^{122\text{-}147}$ and Mis12$^{101\text{-}130}$ fragments have α-helical structure and interact with each other.

Concentration dependence of the circular dichroism spectra of a mixture of Mis12 peptide T_{101}SEE..HLK_{130} and Nnf1 peptide R_{122}FLD…AMA_{147}. Mean residue ellipticity (MRE) values show a double minimum at 208 and 222 nm, characteristic for α-helical structure. Signal becomes stronger with peptide concentration increasing from 13 mM to 100 mM, indicating increased helix content at higher concentration. Concentration dependence implies the presence of intermolecular interactions stabilizing the Mis12-Nnf1 peptide complex. Inset shows the concentration dependence of MRE values at 222 nm for the complex (red) and separate preparation of Mis12 peptide (blue) and Nnf1 (green), indicating the presence of non-

covalent complexes in all three cases in the form of Mis12-Mis12 and Nnf1-Nnf1 dimers and Mis12-Nnf1 dimers and tetramers.

Supplementary Figure S3. NMR of Mis12$^{101-130}$ and Nnf1a$^{122-147}$.

The representative high-field region of 2D[1H-1H]-TOCSY spectra recorded for Mis12 (left) and Nnf1a (right), overlaid with that of equimolar mixture of Mis12 and Nnf1a proteins (green). Signals of V128 and I144 of Nnf1a are assigned unequivocally, while these of Leu residues are only assigned to a separate spin systems (L1-L4). Location of most of resonances, with the exception of a single Leu residue of Nnf1a (L2), visibly varies upon mixing of both protein samples, thus identifying regions involved in the intermolecular interaction.

Supplementary Figure S4. Neither Nnf1a nor Mis12 interacts with CENP-C$^{1-94}$ or Nsl1 on its own.

A. Schematic representation of Y2H experiments used for an analysis of pairwise protein–protein interactions of Mis12 complex subunits (Mis12, Nsl1, Nnf1a) and the amino-terminal region of CENP-C (CENP-C$^{1-188}$). Red arrows indicate positive interactions. Grey arrows with crosses indicate interactions that were tested and gave negative results. Lines with double arrowheads indicate that the interaction was performed in both configurations, meaning each protein was tested in fusion to the transcription activation domain (AD), while the partner was fused to DNA-binding domain (BD), and in the reciprocal fusion protein configuration. As Nsl1 fused to the DNA-binding domain of GAL4 auto-activated the transcription from the promoter, it was not included in the assay. Therefore the interactions with Nsl1 were performed only in a configuration where Nsl1 was fused to AD (indicated by single headed arrows).

B. Western Blots showing results of co-expressions and co-purifications of Mis12 Complex subunits (Mis12, Nsl1, Nnf1a) and the amino-terminal region of CENP-C (CENP-C$^{1-94}$) in

E.coli. Mis12 co-purified with 6xHis::Nnf1a provides positive control. All proteins were expressed as indicated by specific bands in unbound fraction.

Supplementary Figure S5. Size Exclusion Chromatography - Multiple Angle Light Scattering (SEC-MALS) mass measurements of recombinant complexes.

Chromatograms show elution profiles measured in UV_{280} and UV_{254}, Light Scattering (LS) signals and calculated molecular weight (MW). X axis shows elution volume in ml. AU – Arbitrary Unit.

Supplementary Figure S6. SEC-MALS mass measurements of recombinant dimerised complexes

A – C. Chromatograms show elution profiles for 3 reconstituted protein complexes measured in UV_{280} and UV_{254}, Light Scatering (LS) signals and calculated molecular weight (MW). X axis shows elution volume in ml. AU – Arbitrary Unit.

D. Table showing theoretical masses of monomeric and dimeric complexes and masses calculated by Multiple Angle Light Scattering (MALS)

Supplementary Figure 7. Testing the specificity of antibody raised against CENP-C$^{1\text{-}188}$

Western Blot analysis showing specificity of anti-CENP-C$^{1\text{-}188}$ antibody after gene specific RNAi in *D.mel-2* cells.

Bait	Proteins pulled down with bait							
	CENP-C		Nnf1a		Mis12		Nsl1	
	Protein score	Matches	Protein score	Matches	Protein score	Matches	Protein score	Matches
PtA::CENP-C 1-188 aa	4696	93	69	2	423	8	267	4
PtA::CENP-C 1-94 aa	5860	90	243	4	-	-	151	2
GFP::CENP-C	28139	468	1045	24	2715	44	50	1
GFP::CENP-C F26A, F29A	66390	1049	-	-	-	-	-	-
GFP::CENP-C 1-94: F26A, F29A	13122	170	-	-	-	-	-	-
GFP::CENP-C 1-94: L12A, L16A	17934	300	1812	25	852	19	499	6
GFP::Nsl1	-	-	1871	31	5959	145	16153	254
Nsl1 1-101 aa::GFP	-	-	-	-	-	-	17580	233
GFP::Nsl1 102-157aa	16	785	2394	38	8063	130	10404	163
Nsl1 102-157aa::GFP	5	258	1046	23	2211	42	6765	108
GFP::Nsl1 158-183aa	-	-	-	-	-	-	3333	39
GFP::Nsl1 102-183aa	5	185	1273	32	4660	83	6845	95
GFP::Nsl1 102-170aa	1510	23	728	17	3041	54	5751	104
GFP::Nsl1 F138A,T139A,N140A (purification 1)	-	-	-	-	79	2	16689	185
GFP::Nsl1 F138A,T139A,N140A (purification 2)	-	-	133	3	589	15	8299	157
GFP::Nsl1 F138A,T139A,N140A (purification 3)	-	-	58	2	104	3	4984	70
GFP::Nnf1a	541	11	19164	390	1365	37	1386	19
GFP::Nnf1a 122-194 aa	-	-	53164	776	1106	24	172	2
GFP::Nnf1a 1-150 aa	1203	20	33338	457	2959	70	-	-
GFP::Nnf1a L142D	-	-	16554	341	-	-	-	-

GFP:: Nnf1a W41A, I44A, Y45A	-	-	30758	517	1976	44	723	12
GFP::Mis12	1313	28	5335	87	39708	941	4380	71
GFP::Mis12 103-181 aa	-	-	862	18	25809	790	344	5
GFP::Mis12 1-132 aa	433	8	570	16	43650	948	-	-
GFP::Mis12 1-89 aa	-	-	-	-	9357	112	-	-
GFP::Mis12 90-181 aa	-	-	968	18	11854	225	1492	17
GFP::Mis12 L112D, L115D, L126D, L129D	-	-	-	-	24299	421	-	-
GFP::Mis12 L126D, L129D	-	-	-	-	20720	354	-	-
GFP::Mis12 L112D, L115D	-	-	-	-	26491	428		
GFP::Mis12 F12A, F13A, F15A, T16A	-	-	1938	37	46887	908	2292	33

Supplementary Table S1.

List of selected proteins identified in AP-MS experiments. Protein Score is a number that reflects the combined scores of all observed mass spectra that can be matched to amino acid sequences within that protein. Matches reflect number of peptides identified for each protein by Mascot software (Matrix Science). (-) - no matches corresponding to particular protein were identified in the search.

#	AD	BD	Selection	Interaction
1	CENP-C$^{1\text{-}188\ aa}$	Mis12	SD-LW + XαGal	No
2	CENP-C$^{1\text{-}188\ aa}$	Nnf1a	SD-LW + XαGal	No
3	CENP-C$^{1\text{-}188\ aa}$	CENP-C$^{1\text{-}188\ aa}$	SD-LW + XαGal	No
4	CENP-C$^{1\text{-}188\ aa}$	Empty	SD-LW + XαGal	No
5	Empty	CENP-C$^{1\text{-}188\ aa}$	SD-LW + XαGal	No
6	Empty	CENP-C$^{1\text{-}788\ aa}$	SD-LW + Aba	No
7	Mis12	CENP-C$^{1\text{-}788\ aa}$	SD-LW + XαGal	No
8	Nnf1a	CENP-C$^{1\text{-}788\ aa}$	SD-LW + XαGal	No
9	Nsl1	CENP-C$^{1\text{-}788\ aa}$	SD-LW + XαGal	No
10	Empty	CENP-C$^{1\text{-}788\ aa}$	SD-LW + XαGal	No
11	Nnf1a	Mis12	SD-LW + XαGal	Yes
12	Nnf1a	Nnf1a	SD-LW + XαGal	No
13	Nnf1a	CENP-C$^{1\text{-}188\ aa}$	SD-LW + XαGal	No
14	Nnf1a	Empty	SD-LW + XαGal	No
15	Nnf1a	Empty	SD-LW + Aba	No
16	Nnf1a	Mis12 L112D	SD-LW + Aba	No
17	Nnf1a	Mis12 L115D	SD-LW + Aba	No
18	Nnf1a	Mis12 L126D, L129D	SD-LW + Aba	No
19	Nnf1a	Mis12 L126D	SD-LW + Aba	No
20	Nnf1a	Mis12 L129D	SD-LW + Aba	No
21	Empty	Nnf1a	SD-LW + XαGal	No
22	Empty	Nnf1a L142D	SD-LW + Aba	No
23	Mis12	Nnf1a	SD-LW + XαGal	Yes
24	Mis12	Mis12	SD-LW + XαGal	No
25	Mis12	CENP-C$^{1\text{-}188\ aa}$	SD-LW + XαGal	No
26	Mis12	Empty	SD-LW + XαGal	No
27	Mis12	Empty	SD-LW + Aba	No

28	Mis12	Nnf1a L142D	SD-LW + Aba	No
29	Empty	Mis12	SD-LW + XαGal	No
30	Empty	Mis12 L112D	SD-LW + Aba	No
31	Empty	Mis12 L115D	SD-LW + Aba	No
32	Empty	Mis12 L126D, L129D	SD-LW + Aba	No
33	Empty	Mis12 L126D	SD-LW + Aba	No
34	Empty	Mis12 L129D	SD-LW + Aba	No
35	Nsl1	Empty	SD-LW + XαGal	No
36	Nsl1	Empty	SD-LW + Aba	No
37	Empty	Nsl1	SD-LW + XαGal	Yes
38	Nsl1	Nsl1	SD-LW + XαGal	Yes
39	Nsl1	Mis12	SD-LW + XαGal	No
40	Nsl1	Nnf1a	SD-LW + XαGal	No
41	Nsl1	CENP-C$^{1\text{-}188\ aa}$	SD-LW + XαGal	No
42	Nsl1	CENP-C$^{1\text{-}788\ aa}$	SD-LW + Aba	No

Supplementary Table S2.

List of performed Yeast-2-Hybrid experiments. Note that Nsl1 fused to the DNA-binding domain of GAL4 (BD) auto-activated the transcription from the promoter therefore it was not included in further assays. Interactions with Nsl1 were performed only in a configuration where Nsl1 was fused to the transcription activation domain (AD). SD-LW+Aba – selection plate without leucine and tryptophan supplemented with AureobasidinA. SD-LW+ XαGal - selection plate without leucine and tryptophan supplemented with XαGal.

Yeast 2 Hybrid vectors

#	Vector	cDNA	Enzymes used for cloning	Fusion	Selectable marker (for propagation in yeast)	Selectable marker (for propagation in *E.coli*)	Forward primer (used for cloning)	Reverse primer (used for cloning)	Mutagenesis primer - forward	Mutagenesis primer - reverse
1	pGBT9	CenpC$^{1-188}$	EcoR1, BamH1	N terminal Gal4 BD	TRP1	Amp^{R}	5’GTGACAGAATTCATGTCGAACCCCCAGAACAAC3’	5’ATACGCGGATCCTCATTAGGCAACTTCCTCTTTTTGTTT3’	----	----
2	pGBT9	Mis12	EcoR1, Sal1	N terminal Gal4 BD	TRP1	Amp^{R}	5’GTGACAGAATTCATGGACTTCAATAGCCTAGCC3’	5’ATACGCGTCGACTCATTAATCAGTCTCCTTCTTTATGTC3’	----	----
3	pGBT9	Nnf1a	Sal1, Sma1	N terminal Gal4 BD	TRP1	Amp^{R}	5’GTGACACCCGGGTATGGAGGATTCGGAAGCC3’	5’ATACGCGTCGACTCATTAGAAGTCGTTCAATGCTTCGCC3’	----	----
4	pGBT9	Nsl1	EcoR1, Sal1	N terminal Gal4 BD	TRP1	Amp^{R}	5’GTGACAGAATTCATGGAGCCAGCCGAAAGTCCA3’	5’ATACGCGTCGACTCATTACCGTTGGTTGGCCATATTCTG3’	----	----
5	pGAD424	CenpC$^{1-188}$	EcoR1, BamH1	N terminal Gal4 AD	LEU2	Amp^{R}	5’GTGACAGAATTCATGTCGAACCCCCAGAACAAC3’	5’ATACGCGGATCCTCATTAGGCAACTTCCTCTTTTTGTTT3’	----	----
6	pGAD424	Mis12	EcoR1, Sal1	N terminal Gal4 AD	LEU2	Amp^{R}	5’GTGACAGAATTCATGGACTTCAATAGCCTAGCC3’	5’ATACGCGTCGACTCATTAATCAGTCTCCTTCTTTATGTC3’	----	----
7	pGAD424	Nnf1a	Sal1, Sma1	N terminal Gal4 AD	LEU2	Amp^{R}	5’GTGACACCCGGGTATGGAGGATTCGGAAGCC3’	5’ATACGCGTCGACTCATTAGAAGTCGTTCAATGCTTCGCC3’	----	----
8	pGAD424	Nsl1	EcoR1, Sal1	N terminal Gal4 AD	LEU2	Amp^{R}	5’GTGACAGAATTCATGGAGCCAGCCGAAAGTCCA3’	5’ATACGCGTCGACTCATTACCGTTGGTTGGCCATATTCTG3’	----	----
9	pGBKT7	CenpC$^{1-788\ aa}$	Nde1, BamH1	N terminal Gal4 BD	TRP1	Kan^{R}	5’AGGAGGACCTGCATATGATGGTCGAAGCCC3’	5’TAGGATCCCTACAGTTCGTTCTCCATCGCC3’	----	----
10	pGBT9	CenpC$^{1-788\ aa}$	EcoR1, BamH1	N terminal Gal4 BD	TRP1	Amp^{R}	5’GTGACAGAATTCATGTCGAAGCCCCAGAACAAC3’	5’ATACGCGGATCCTCATTACAGTTCGTTCTCCATCGCCCT3’	----	----
11	pGBT9	Mis12^{L112D}	EcoR1, Sal1	N terminal Gal4 BD	TRP1	Amp^{R}	5’GTGACAGAATTCATGGACTTCAATAGCCTAGCC3’	5’ATACGCGTCGACTCATTAATCAGTCTCCTTCTTTATGTC3’	5’AGAGGAGGAGCAGAAGACAGCCAGGGACGAGGAGCTGAAGGCCAAATACAGAGAGAACATGG3’	5’CCATGTTCTCTCTGTATTTGGCCTTCAGCTCCTCGTCCCTGGCTGTCTTCTGCTCCTCCTCT3’
12	pGBT9	Mis12^{L115D}	EcoR1, Sal1	N terminal Gal4 BD	TRP1	Amp^{R}	5’GTGACAGAATTCATGGACTTCAATAGCCTAGCC3’	5’ATACGCGTCGACTCATTAATCAGTCTCCTTCTTTATGTC3’	5’AGAGGAGGAGCAGAAGACAGCCAGGTTGGAGGAGGACAAGGCCAAATACAGAGAGAACATGG3’	5’CCATGTTCTCTCTGTATTTGGCCTTGTCCTCCTCCAACCTGGCTGTCTTCTGCTCCTCCTCT3’
13	pGBT9	Mis12^{L126D,L129D}	EcoR1, Sal1	N terminal Gal4 BD	TRP1	Amp^{R}	5’GTGACAGAATTCATGGACTTCAATAGCCTAGCC3’	5’ATACGCGTCGACTCATTAATCAGTCTCCTTCTTTATGTC3’	5’CAAATACAGAGAGAACATGGCCATGGACGCGCATGACAAGATCGAGGAGGAGAAGTACGCCG3’	5’CGGCGTACTTCTCCTCCTCGATCTTGTCATGCGCGTCCATGGCCATGTTCTCTCTGTATTTG3’
14	pGBT9	Mis12^{L126D}	EcoR1, Sal1	N terminal Gal4 BD	TRP1	Amp^{R}	5’GTGACAGAATTCATGGACTTCAATAGCCTAGCC3’	5’ATACGCGTCGACTCATTAATCAGTCTCCTTCTTTATGTC3’	5’CAAATACAGAGAGAACATGGCCATGGACGCGCATTTGAAGATCGAGGAGGAGAAGTACGCCG3’	5’CGGCGTACTTCTCCTCCTCGATCTTCAAATGCGCGTCCATGGCCATGTTCTCTCTGTATTTG3’
15	pGBT9	Mis12^{L129D}	EcoR1, Sal1	N terminal Gal4 BD	TRP1	Amp^{R}	5’GTGACAGAATTCATGGACTTCAATAGCCTAGCC3’	5’ATACGCGTCGACTCATTAATCAGTCTCCTTCTTTATGTC3’	5’CAAATACAGAGAGAACATGGCCATGCTGGCGCATGACAAGATCGAGGAGGAGAAGTACGCCG3’	5’CGGCGTACTTCTCCTCCTCGATCTTGTCATGCGCCAGCATGGCCATGTTCTCTCTGTATTTG3’
16	pGBT9	Nnf1a^{L142D}	Sal1, Sma1	N terminal Gal4 BD	TRP1	Amp^{R}	5’GTGACACCCGGGTATGGAGGATTCGGAAGCC3’	5’ATACGCGTCGACTCATTAGAAGTCGTTCAATGCTTCGCC3’	5’CAGCGTGGAATTCATGGAGCAGCAACTGGCCTCTCAGGCAAAAGAAGATGAGATTGCTATGGCCAAGAGCAAT3’	5’ATTGCTCTTGGCCATAGCAATCTCATCTTCTTTTGCCTGAGAGGCCAGTTGCTGCTCCATGAATTCCACGCTG3’

Gateway expression vectors

#	Vector	cDNA	Fusion	Selectable marker (for propagation in *E.coli*)	Forward primer (used for generation of entry clone)	Reverse primer (used for generation of entry clone)	Mutagenesis primer - forward	Mutagenesis primer - reverse
1	pMT-GFP	CenpC	N terminal eGFP	Amp^R	5’GGGGACAAGTTTGTACAAAAAAGCAGGCTTAATGTCGAAGCCCCAGAACAACGACA3’	5’GGGGACCACTTTGTACAAGAAAGCTGGGTACTAACTGCGTATACACATCAGCACA3’	----	----
2	pMT-GFP	CenpC F26A, F29A	N terminal eGFP	Amp^R	5’GGGGACAAGTTTGTACAAAAAAGCAGGCTTAATGTCGAAGCCCCAGAACAACGACA3’	5’GGGGACCACTTTGTACAAGAAAGCTGGGTACTAACTGCGTATACACATCAGCACA3’	5’CCAGCCAGTGAAGGACAAGGAGCGCGCCGCCGCCGCCATGATGCGCAAGCTTGCCGAAAATA3’	5’TATTTTCGGCAAGCTTGCGCATCATGGCGGCGGCGGCGCGCTCCTTGTCCTTCACTGGCTGG3’
3	pMT-GFP	CenpC $^{1-94\ F26A,\ F29A}$	N terminal eGFP	Amp^R	5’GGGGACAAGTTTGTACAAAAAAGCAGGCTTAATGTCGAAGCCCCAGAACAACGAC3’	5’GGGGACCACTTTGTACAAGAAAGCTGGGTACTAATTGACTTTCTCGGTGGCGGC3’	5’CCAGCCAGTGAAGGACAAGGAGCGCGCCGCCGCCGCCATGATGCGCAAGCTTGCCGAAAATA3’	5’TATTTTCGGCAAGCTTGCGCATCATGGCGGCGGCGGCGCGCTCCTTGTCCTTCACTGGCTGG3’
4	pMT-GFP	CenpC $^{1-94\ L12A,\ L16A}$	N terminal eGFP	Amp^R	5’GGGGACAAGTTTGTACAAAAAAGCAGGCTTAATGTCGAAGCCCCAGAACAACGAC3’	5’GGGGACCACTTTGTACAAGAAAGCTGGGTACTAATTGACTTTCTCGGTGGCGGC3’	5’GCCCCAGAACAACGACACTCTGGAGGCCGACGACATCGCCAGCCAGCCAGTGAAGGACAAGGAGC3’	5’GCTCCTTGTCCTTCACTGGCTGGCTGGCGATGTCGTCGGCCTCCAGAGTGTCGTTGTTCTGGGGC3’
5	pMT-DESTNPta	CenpC $^{1-188}$	N terminal Protein A	Amp^R	5’GGGGACAAGTTTGTACAAAAAAGCAGGCTTAATGTCGAAGCCCCAGAACAACG3’	5’GGGGACCACTTTGTACAAGAAAGCTGGGTACTAGGCAACTTCCTCTTTTTGT3’	----	----
6	pMT-DESTNPta	CenpC $^{1-94}$	N terminal Protein A	Amp^R	5’GGGGACAAGTTTGTACAAAAAAGCAGGCTTAATGTCGAAGCCCCAGAACAACGAC3’	5’GGGGACCACTTTGTACAAGAAAGCTGGGTACTAATTGACTTTCTCGGTGGCGGC3’	----	----
7	pMT-GFP	Nsl1	N terminal eGFP	Amp^R	5’GGGGACAAGTTTGTACAAAAAAGCAGGCTTAATGGAGCCAGCCGAAAGTCCAGAAA3’	5’GGGGACCACTTTGTACAAGAAAGCTGGGTATCACCGTTGGTTGGCCATATTCTGG3’	----	----
8	pMT-GFP	Nsl1$^{1-101}$	C terminal eGFP	Amp^R	5’GGGGACAAGTTTGTACAAAAAAGCAGGCTTAATGGAGCCAGCCGAAAGTCCAGAAA3’	5’GGGGACCACTTTGTACAAGAAAGCTGGGTACGCATGCATTACAAAGTCGAAGAAC3’	----	----
9	pMT-GFP	Nsl1$^{102-183}$	N terminal eGFP	Amp^R	5’GGGGACAAGTTTGTACAAAAAAGCAGGCTTAGCACTGGACACGGACAATCGCAAGG3’	5’GGGGACCACTTTGTACAAGAAAGCTGGGTATTATCACCGTTGGTTGGCCATATTC3’	----	----
10	pMT-GFP	Nsl1$^{102-157}$	C terminal eGFP	Amp^R	5’GGGGACAAGTTTGTACAAAAAAGCAGGCTTAATGGCACTGGACACGGACAATCGCA3’	5’GGGGACCACTTTGTACAAGAAAGCTGGGTAGCACTGCAGCTCCATCATGTCCTCG3’	----	----
11	pMT-GFP	Nsl1$^{102-157}$	N terminal eGFP	Amp^R	5’GGGGACAAGTTTGTACAAAAAAGCAGGCTTAGCACTGGACACGGACAATCGCAAGG3’	5’GGGGACCACTTTGTACAAGAAAGCTGGGTATTAGCACTGCAGCTCCATCATGTCC3’	----	----

12	pMT-GFP	Nsl1$^{158-183}$	N terminal eGFP	Amp^{R}	5’GGGGACAAGTTTGTACAAAAAAGCAGGCTTAAATATGGACGATCACTACCTATTCA3’	5’GGGGACCACTTTGTACAAGAAAGCTGGGTATTATCACCGTTGGTTGGCCATATTC3’	----	----
13	pMT-GFP	Nsl1$^{158-170}$	N terminal eGFP	Amp^{R}	5’GGGGACAAGTTTGTACAAAAAAGCAGGCTTAAATATGGACGATCACTACCTATTCA3’	5’GGGGACCACTTTGTACAAGAAAGCTGGGTATTACATGGTGTTCCGCATGAATAGGTAG3’	----	----
14	pMT-GFP	Nsl1 L136A	N terminal eGFP	Amp^{R}	5’GGGGACAAGTTTGTACAAAAAAGCAGGCTTAATGGAGCCAGCCGAAAGTCCAGAAA3’	5’GGGGACCACTTTGTACAAGAAAGCTGGGTATCACCGTTGGTTGGCCATATTCTGG3’	5’TTTGCAAAACTTCGTGCGAAGCTCGGCGGCTTTCACAAACGCGGCCATCCGAT3’	5’ATCGGATGGCCGCGTTTGTGAAAGCCGCCGAGCTTCGCACGAAGTTTTGCAAA3’
15	pMT-GFP	Nsl1 $^{F138A, T139A, N140A}$	N terminal eGFP	Amp^{R}	5’GGGGACAAGTTTGTACAAAAAAGCAGGCTTAATGGAGCCAGCCGAAAGTCCAGAAA3’	5’GGGGACCACTTTGTACAAGAAAGCTGGGTATCACCGTTGGTTGGCCATATTCTGG3’	5’AAAACTTCGTGCGAAGCTCGTTGGCTGCCGCCGCCGCGGCCATCCGATTTCAGGCGGAGC3’	5’GCTCCGCCTGAAATCGGATGGCCGCGGCGGCGGCAGCCAACGAGCTTCGCACGAAGTTTT3’
16	pMT-GFP	Nnf1a	N terminal eGFP	Amp^{R}	5’GGGGACAAGTTTGTACAAAAAAGCAGGCTTAATGGAGGATTCGGAAGCCGCATTTA3’	5’GGGGACCACTTTGTACAAGAAAGCTGGGTATCAGAAGTCGTTCAATGCTTCGCCT3’	----	----
17	pMT-GFP	Nnf1a$^{122-194}$	N terminal eGFP	Amp^{R}	5’GGGGACAAGTTTGTACAAAAAAGCAGGCTTACGATTTTTGGACTTCAGCGTGGAAT3’	5’GGGGACCACTTTGTACAAGAAAGCTGGGTATTATCAGAAGTCGTTCAATGCTTCG3’	----	----
18	pMT-GFP	Nnf1a$^{1-150}$	N terminal eGFP	Amp^{R}	5’GGGGACAAGTTTGTACAAAAAAGCAGGCTTAGAGGATTCGGAAGCCGCATTTAAA3’	5’GGGGACCACTTTGTACAAGAAAGCTGGGTATCAATTGCTCTTGGCCATAGCAATCT3’	----	----
19	pMT-GFP	Nnf1a ^{L142D}	N terminal eGFP	Amp^{R}	5’GGGGACAAGTTTGTACAAAAAAGCAGGCTTAATGGAGGATTCGGAAGCCGCATTTA3’	5’GGGGACCACTTTGTACAAGAAAGCTGGGTATCAGAAGTCGTTCAATGCTTCGCCT3’	5’CAGCGTGGAATTCATGGAGCAGCAACTGGCCTCTCAGGCAAAAGAAGATGAGATTGCTATGGCCAAGAGCAAT3’	5’ATTGCTCTTGGCCATAGCAATCTCATCTTCTTTTGCCTGAGAGGCCAGTTGCTGCTCCATGAATTCCACGCTG3’
20	pMT-GFP	Nnf1a $^{W41A, I44A, Y45A}$	N terminal eGFP	Amp^{R}	5’GGGGACAAGTTTGTACAAAAAAGCAGGCTTAATGGAGGATTCGGAAGCCGCATTTA3’	5’GGGGACCACTTTGTACAAGAAAGCTGGGTATCAGAAGTCGTTCAATGCTTCGCCT3’	5’GCTGCGCAGATCTGCAAGCGGCGGATGCCGCTGCCCAGGAGCACGAGCAATCCGCTCTGG3’	5’CCAGAGCGGATTGCTCGTGCTCCTGGGCAGCGGCATCCGCCGCTTGCAGATCTGCGCAGC3’
21	pMT-GFP	Mis12	N terminal eGFP	Amp^{R}	5’GGGGACAAGTTTGTACAAAAAAGCAGGCTTAATGGACTTCAATAGCCTAGCCTACG3’	5’GGGGACCACTTTGTACAAGAAAGCTGGGTATTAATCAGTCTCCTTCTTTATCTGC3’	----	----
22	pMT-GFP	Mis12$^{103-181}$	N terminal eGFP	Amp^{R}	5’GGGGACAAGTTTGTACAAAAAAGCAGGCTTAGAAGAGGAGGAGCAGAAGACAGCCA3’	5’GGGGACCACTTTGTACAAGAAAGCTGGGTATTAATCAGTCTCCTTCTTTATCTGC3’	----	----
23	pMT-GFP	Mis12$^{1-132}$	N terminal eGFP	Amp^{R}	5’GGGGACAAGTTTGTACAAAAAAGCAGGCTTAGACTTCAATAGCCTAGCCTACGATC3’	5’GGGGACCACTTTGTACAAGAAAGCTGGGTATTACTCGATCTTCAAATGCGCCAGC3’	----	----
24	pMT-GFP	Mis12$^{1-89}$	N terminal eGFP	Amp^{R}	5’GGGGACAAGTTTGTACAAAAAAGCAGGCTTAATGGACTTCAATAGCCTAGCCTACG3’	5’GGGGACCACTTTGTACAAGAAAGCTGGGTACAGCACATGCGGTGGAACGTGGAAC3’	----	----
25	pMT-GFP	Mis12$^{90-181}$	N terminal eGFP	Amp^{R}	5’GGGGACAAGTTTGTACAAAAAAGCAGGCTTACATCCGGAGCACATGTTCGTCGAGA3’	5’GGGGACCACTTTGTACAAGAAAGCTGGGTATTAATCAGTCTCCTTCTTTATCTGC3’	----	----
26	pMT-GFP	Mis12 $^{L112D, L115D, L126D, L129D}$	N terminal eGFP	Amp^{R}	5’GGGGACAAGTTTGTACAAAAAAGCAGGCTTAATGGACTTCAATAGCCTAGCCTACG3’	5’GGGGACCACTTTGTACAAGAAAGCTGGGTATTAATCAGTCTCCTTCTTTATCTGC3’	L112D, L115D:<br>5’AGAGGAGGAGCAGAAGACAGCCAGGGACGAGGAGGACAAGGCCAAATACAGAGAGAACATGG3’	L112D, L115D:<br>5’CCATGTTCTCTCTGTATTTGGCCTTGTCCTCCTCGTCCCTGGCTGTCTTCTGCTCCTCCTCT3’

							L126D, L129D: 5'CAAATACAGAGAGAACATGGCCATGGACGCGCATGACAAGATCGAGGAGGAGAAGTACGCCG3'	L126D, L129D: 5'CGGCGTACTTCTCCTCCTCGATCTTGTCATGCGCGTCCATGGCCATGTTCTCTCTGTATTTG3'
27	pMT-GFP	Mis12 $^{L126D, L129D}$	N terminal eGFP	Amp^{R}	5'GGGGACAAGTTTGTACAAAAAAGCAGGCTTAATGGACTTCAATAGCCTAGCCTACG3'	5'GGGGACCACTTTGTACAAGAAAGCTGGGTATTAATCAGTCTCCTTCTTTATCTGC3'	5'CAAATACAGAGAGAACATGGCCATGGACGCGCATGACAAGATCGAGGAGGAGAAGTACGCCG3'	5'CGGCGTACTTCTCCTCCTCGATCTTGTCATGCGCGTCCATGGCCATGTTCTCTCTGTATTTG3'
28	pMT-GFP	Mis12 $^{L112D, L115D}$	N terminal eGFP	Amp^{R}	5'GGGGACAAGTTTGTACAAAAAAGCAGGCTTAATGGACTTCAATAGCCTAGCCTACG3'	5'GGGGACCACTTTGTACAAGAAAGCTGGGTATTAATCAGTCTCCTTCTTTATCTGC3'	5'AGAGGAGGAGCAGAAGACAGCCAGGGACGAGGAGGACAAGGCCAAATACAGAGAGAACATGG3'	5'CCATGTTCTCTCTGTATTTGGCCTTGTCCTCCTCGTCCCTGGCTGTCTTCTGCTCCTCCTCT3'
29	pMT-GFP	Mis12 $^{F12A, F13A, F15A, T16A}$	N terminal eGFP	Amp^{R}	5'GGGGACAAGTTTGTACAAAAAAGCAGGCTTAATGGACTTCAATAGCCTAGCCTACG3'	5'GGGGACCACTTTGTACAAGAAAGCTGGGTATTAATCAGTCTCCTTCTTTATCTGC3'	5'CAATAGCCTAGCCTACGATCAGAAGGCCGCCAATGCCGCCGCGGCACAGTTGTCTGCAGAGCGCG3'	5'CGCGCTCTGCAGACAACTGTGCCGCGGCGGCATTGGCGGCCTTCTGATCGTAGGCTAGGCTATTG3'

Duet Vectors

#	Vector	MCSI			MCSII		Selectable marker (for propagation in *E.coli*)	Forward primer MCSI (used for cloning)	Reverse primer MCSI (used for cloning)	Forward primer MCSII (used for cloning)	Reverse primer MCSII (used for cloning)	Mutagenesis primer - forward	Mutagenesis primer - reverse
		cDNA	Enzymes used for cloning	His tag	cDNA	Enzymes used for cloning							
1	pET	Mis12	BamH1, Asc1	+	----	----	Amp^{R}	5'CAGGATCCTATGGACTTCAATAGCCTA3'	5'TGGCGCGCCCTATTAATCAGTCTCCTTCTT3'	----	----	----	----
2	pET	Nsl1	EcoR1, Asc1	+	----	----	Amp^{R}	5'CGAATTCGATGGAGCCAGCCGAAAGT3'	5'TGGCGCGCCTTATCACCGTTGGTTGGCCAT3'	----	----	----	----
3	pET	Mis12	BamH1, Asc1	+	Nsl1	Bgl2, Kpn1	Amp^{R}	5'CAGGATCCTATGGACTTCAATAGCCTA3'	5'TGGCGCGCCCTATTAATCAGTCTCCTTCTT3'	5'GAAGATCTCATGGAGCCAGCCGAAAGT3'	5'GTGGTACCTTATCACCGTTGGTTGGCCAT3'	----	----
4	pET	----	----	-	Nsl1	Bgl2, Kpn1	Amp^{R}	----	----	5'GAAGATCTCATGGAGCCAGCCGAAAGT3'	5'GTGGTACCTTATCACCGTTGGTTGGCCAT3'	----	----
5	pCOLA	Nnf1a	BamH1, Asc1	+	----	----	Kan^{R}	5'CGGGATCCTATGGAGGATTCGGAAGCC3'	5'AGGCGCGCCTCAGAAGTCGTTCAATGC3'	----	----	----	----
6	pCOLA	Nnf1a	BamH1, Asc1	+	Mis12	Nde1, Asis1	Kan^{R}	5'CGGGATCCTATGGAGGATTCGGAAGCC3'	5'AGGCGCGCCTCAGAAGTCGTTCAATGC3'	5'GTGACACATATGATGGACTTCAATAGCCTA3'	5'ATACGCGCGATCGCTCATTAATCAGTCTCCTTCTTTAT3'	----	----
7	pCOLA	Nnf1a	Nco1, BamH1	-	Mis12	Nde1, Asis1	Kan^{R}	5'CCTTCCATGGTTATGGAGGATTCGGAAGCC3'	5'CGGGATCCTTATCAGAAGTCGTTCAATGC3'	5'GTGACACATATGATGGACTTCAATAGCCTA3'	5'ATACGCGCGATCGCTCATTAATCA	----	----

										GTCTCCTTCTTTAT3’		
8	pACYC	CenpC$^{1-94}$	BamH1, Asc1	+	----	----	Cm^R	5’GTGACAGGATCCTTCGAAGCCCCAGAACAACGAC3’	5’ATACGCGGCGCGCCTCATTAGACTTTCTCGGTGGCGGCACTGTTTTT3’	----	----	----	----
9	pACYC	CenpC$^{1-94}$	Nco1, Asc1	-	----	----	Cm^R	5’GTGACACCATGGGCATGTCGAAGCCCCAGAACAAC3’	5’ATACGCGGCGCGCCTCATTAGACTTTCTCGGTGGCGGCACTGTTTTT3’	----	----	----	----
10	pACYC	CenpC$^{1-94\ F26A,F29A}$	BamH1, Asc1	+	----	----	Cm^R	5’GTGACAGGATCCTTCGAAGCCCCAGAACAACGAC3’	5’ATACGCGGCGCGCCTCATTAGACTTTCTCGGTGGCGGCACTGTTTTT3’	----	----	5’CCAGCCAGTGAAGGACAAGGAGCGCGCCGCCGCCGCCATGATGCGCAAGCTTGCCGAAAATA3’	5’TATTTTCGGCAAGCTTGCGCATCATGGCGGCGGCGGCGCGCTCCTTGTCCTTCACTGGCTGG3’
11	pCOLA	Nnf1a	BamH1, Asc1	+	Mis12 $^{F12A,\ F13A,\ F15A,\ T16A}$	Nde1, Asis1	Kan^R	5’CGGGATCCTATGGAGGATTCGGAAGCC3’	5’AGGCGCGCCTCAGAAGTCGTTCAATGC3’	5’GTGACACATATGATGGACTTCAATAGCCTA3’	5’ATACGCGCGATCGCTCATTAATCAGTCTCCTTCTTTAT3’	5’CAATAGCCTAGCCTACGATCAGAAGGCCGCCAATGCCGCCGCGGCACAGTTGTCTGCAGAGCGCG3’	5’CGCGCTCTGCAGACAACTGTGCCGCGGCGGCATTGGCGGCCTTCTGATCGTAGGCTAGGCTATTG3’
12	pCOLA	Nnf1a$^{W41A,\ I44A,\ Y45A}$	BamH1, Asc1	+	Mis12	Nde1, Asis1	Kan^R	5’CGGGATCCTATGGAGGATTCGGAAGCC3’	5’AGGCGCGCCTCAGAAGTCGTTCAATGC3’	5’GTGACACATATGATGGACTTCAATAGCCTA3’	5’ATACGCGCGATCGCTCATTAATCAGTCTCCTTCTTTAT3’	5’GCTGCGCAGATCTGCAAGCGGCGGATGCCGCTGCCCAGGAGCACGAGCAATCCGCTCTGG3’	5’CCAGAGCGGATTGCTCGTGCTCCTGGGCAGCGGCATCCGCCGCTTGCAGATCTGCGCAGC3’
13	pCOLA	Nnf1a$^{122-196}$	BamH1, Asc1	+	Mis12	Nde1, Asis1	Kan^R	5’GTGACAGGATCCTCGCATGCGATTTTTGGACTTCAGCGTG3’	5’ATACGCGGCGCGCCTCATTAGAAGTCGTTCAATGCTTCGCCTAGT3’	5’GTGACACATATGATGGACTTCAATAGCCTA3’	5’ATACGCGCGATCGCTCATTAATCAGTCTCCTTCTTTAT3’	----	----
14	pCOLA	Nnf1a$^{122-196}$	BamH1, Asc1	+	Mis12$^{104-181}$	Nde1, Asis1	Kan^R	5’GTGACAGGATCCTCGCATGCGATTTTTGGACTTCAGCGTG3’	5’ATACGCGGCGCGCCTCATTAGAAGTCGTTCAATGCTTCGCCTAGT3’	5’GTGACACATATGATGGAAGAGGAGGAGCAGAAGACAGCC3’	5’ATACGCGCGATCGCTCATTAATCAGTCTCCTTCTTTAT3’	----	----
15	pCOLA	Nnf1a	BamH1, Asc1	+	Mis12$^{104-181}$	Nde1, Asis1	Kan^R	5’CGGGATCCTATGGAGGATTCGGAAGCC3’	5’AGGCGCGCCTCAGAAGTCGTTCAATGC3’	5’GTGACACATATGATGGAAGAGGAGGAGCAGAAGACAGCC3’	5’ATACGCGCGATCGCTCATTAATCAGTCTCCTTCTTTAT3’	----	----

Supplementary Table S3.

List of vectors used in this study with sequences of primers used for cloning and mutagenesis.

Supplementary References

51. Mason JM, Arndt KM 2004 Coiled coil domains: stability, specificity, and biological implications. Chembiochem 5: 170–6. doi:10.1002/cbic.200300781

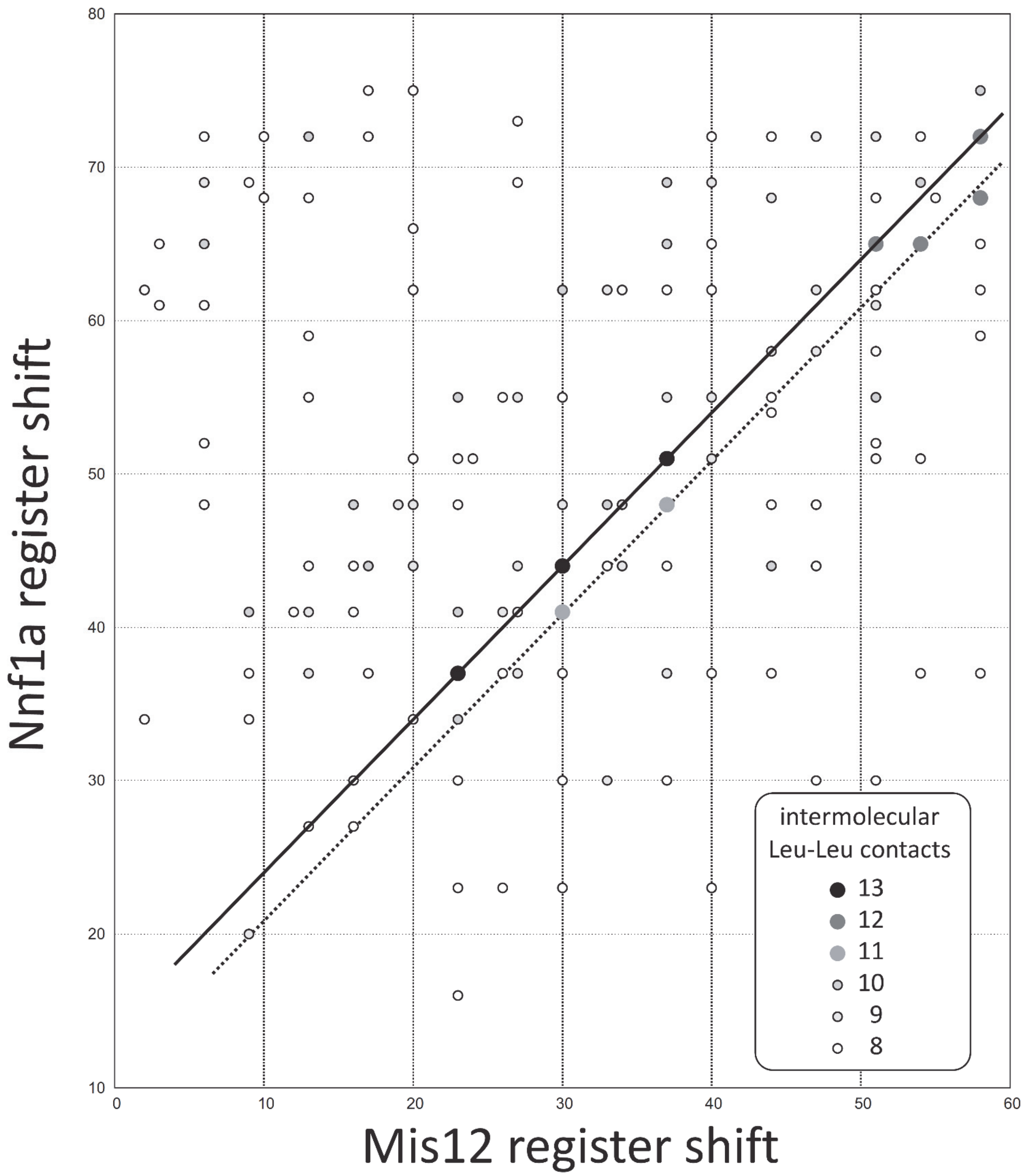

Supplementary Figure S1

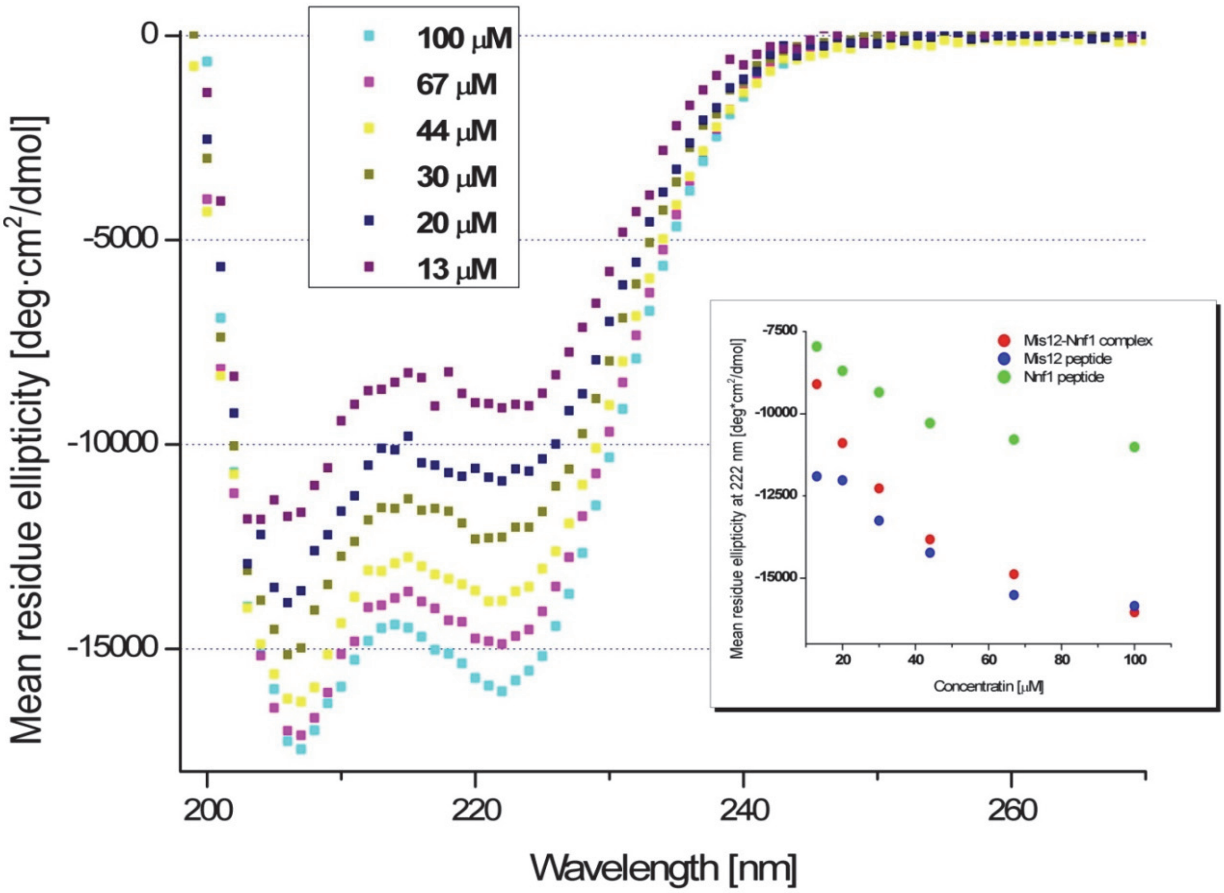

Supplementary Figure S2

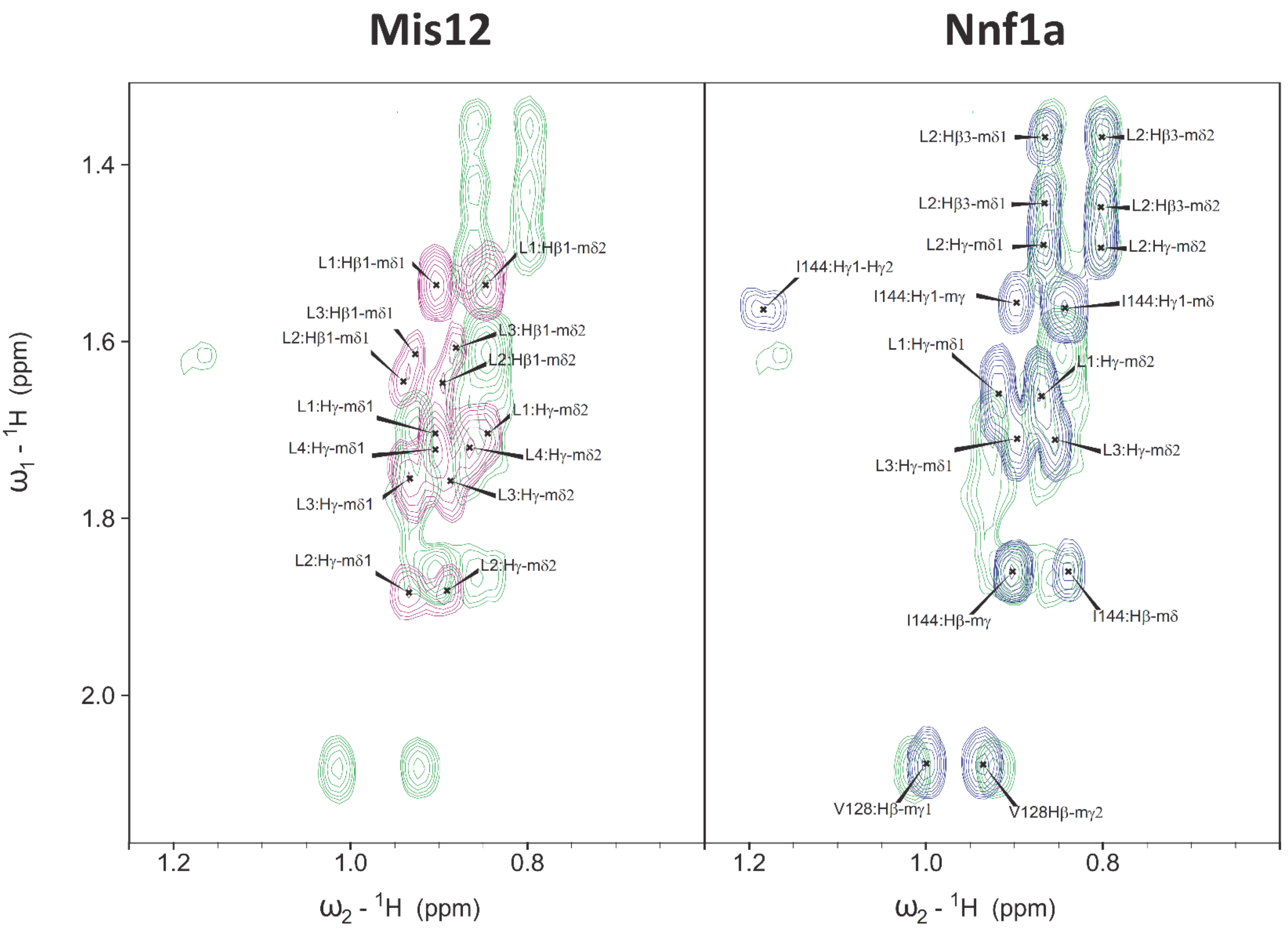

Supplementary Figure S3

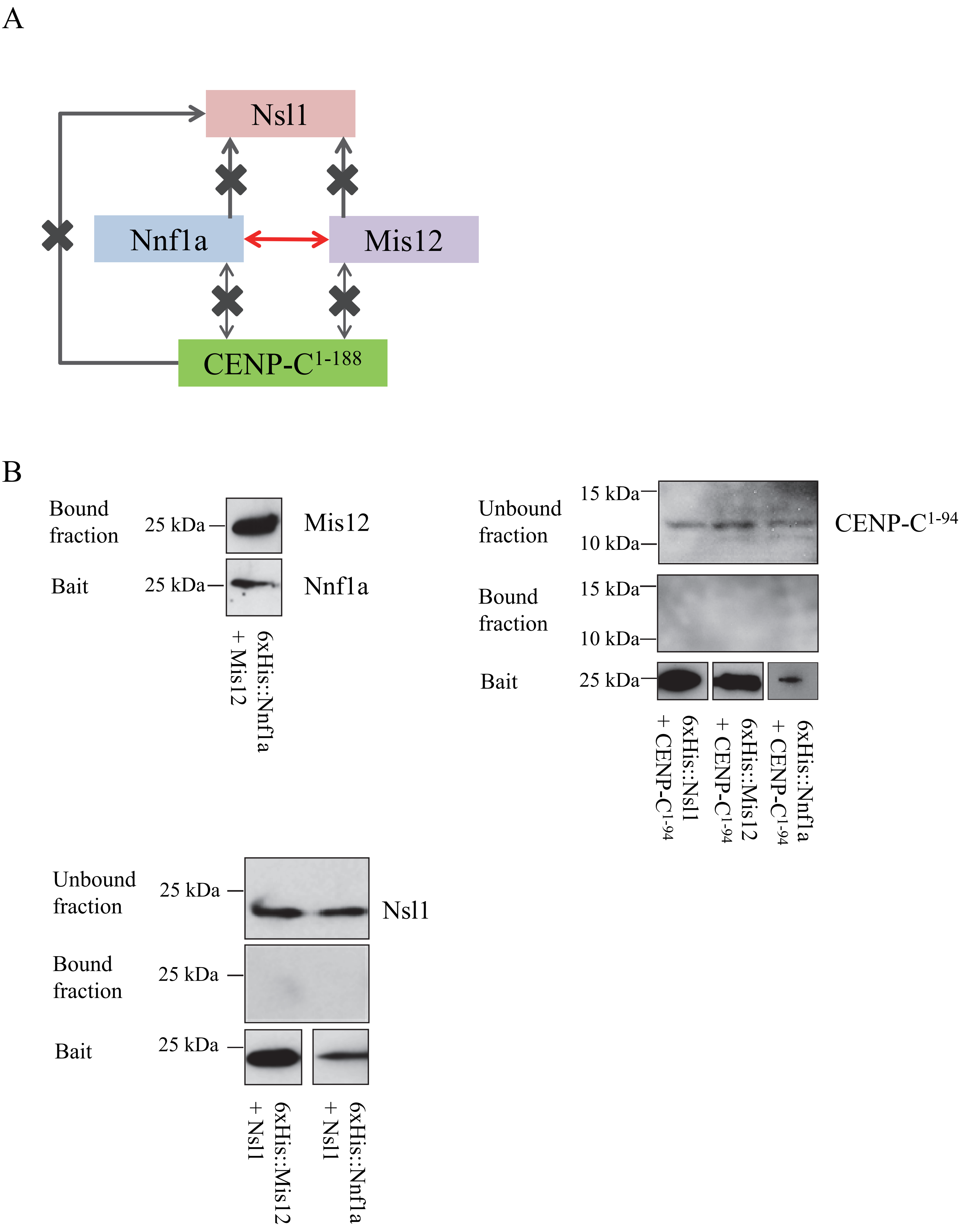

Supplementary Figure S4

A

6xHis::Nnf1a+Mis12

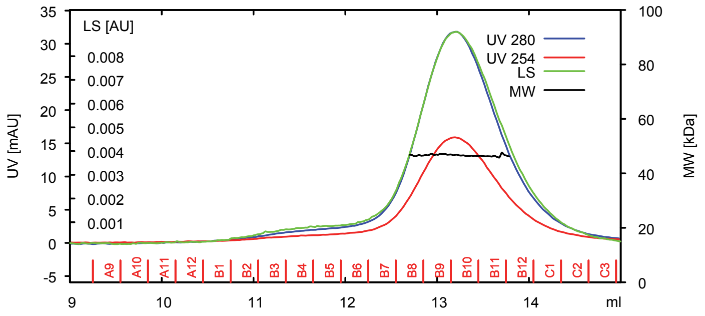

Mw: 46 kDa

B

6xHis::CENP-C$^{1-94}$+Nnf1a+Mis12

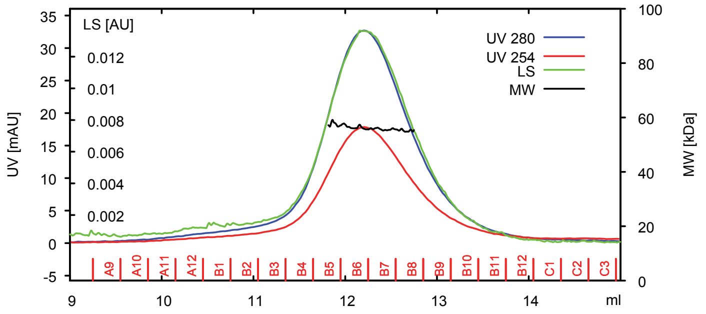

Mw: 56 kDa

C

6xHis::Nnf1a+Mis12+CENP-C$^{1-94}$

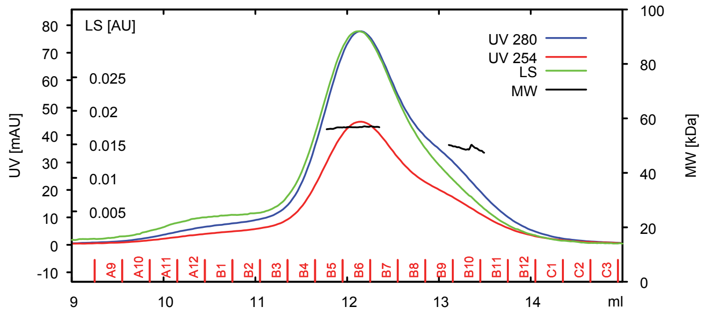

Mw: 56 kDa

D

6xHis::Nsl1+Nnf1a+Mis12

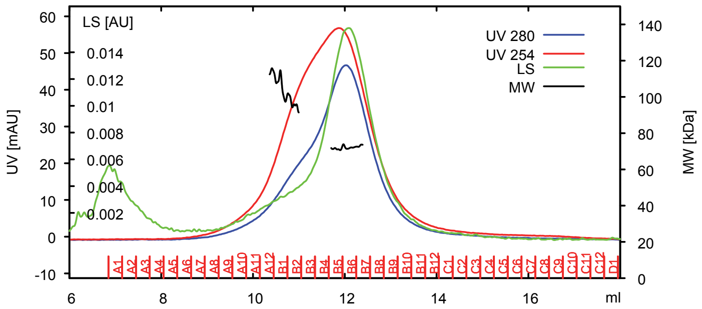

Mw: 72 kDa

Supplementary Figure S5

A

6xHis::Nnf1a+Mis12

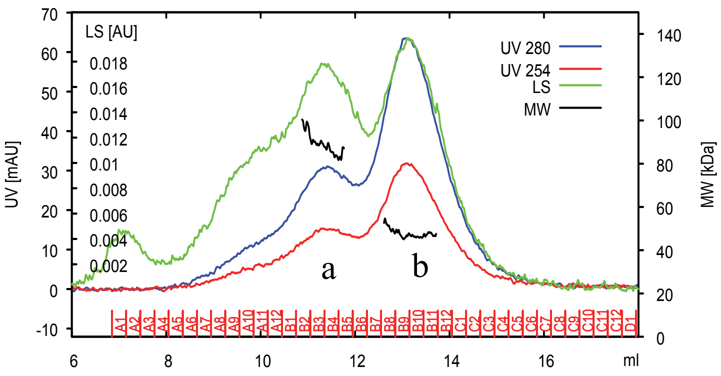

a) Mw: 88 kDa a) Mw: 47 kDa

B

6xHis::CENP-C$^{1-94}$+Nnf1a+Mis12

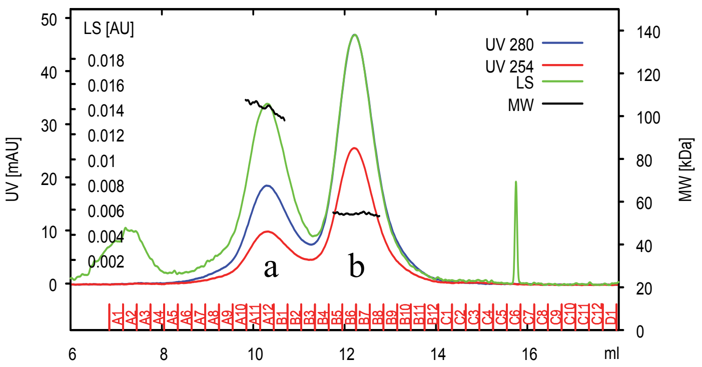

a) Mw: 103 kDa a) Mw: 54 kDa

C

6xHis::Nnf1a+Mis12+CENP-C$^{1-94}$

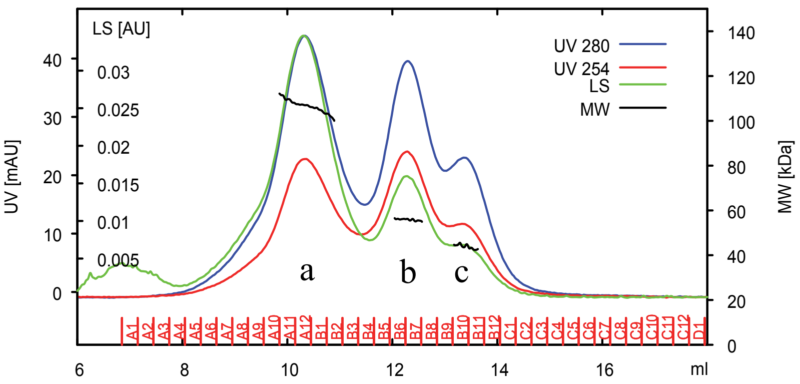

a) Mw: 106 kDa b) Mw: 56 kDa c) Mw: 44 kDa

D

Complex	Theoretical mass of monomeric complex (kDa)	Theoretical mass of dimeric complex (kDa)	Mass calculated by MALS (kDa)
6xHis::Nnf1a+Mis12	45.378	90.75	88.79
6xHis::Nnf1a+Mis12 + CENP-C$^{1-94}$	56.367	112.73	106.6
6xHis:: CENP-C$^{1-94}$ + Nnf1a + Mis12	56.525	113.05	103.7

Supplementary Figure S6

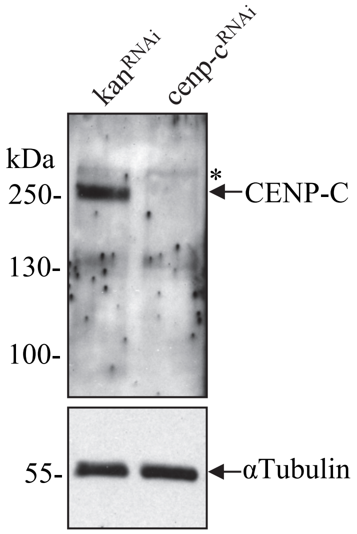

Supplementary Figure S7
